# Final Report of the Intergroup Randomized Study of Combined Androgen-Deprivation Therapy Plus Radiotherapy Versus Androgen-Deprivation Therapy Alone in Locally Advanced Prostate Cancer

**DOI:** 10.1200/JCO.2014.57.7510

**Published:** 2015-02-17

**Authors:** Malcolm D. Mason, Wendy R. Parulekar, Matthew R. Sydes, Michael Brundage, Peter Kirkbride, Mary Gospodarowicz, Richard Cowan, Edmund C. Kostashuk, John Anderson, Gregory Swanson, Mahesh K.B. Parmar, Charles Hayter, Gordana Jovic, Andrea Hiltz, John Hetherington, Jinka Sathya, James B.P. Barber, Michael McKenzie, Salah El-Sharkawi, Luis Souhami, P.D. John Hardman, Bingshu E. Chen, Padraig Warde

**Affiliations:** Malcolm D. Mason, Cardiff University School of Medicine, Velindre Hospital; James B.P. Barber, Velindre Hospital, Cardiff; Matthew R. Sydes, Mahesh K.B. Parmar, Gordana Jovic, Medical Research Council Clinical Trials Unit at University College London, London; Peter Kirkbride, The Clatterbridge Cancer Centre National Health Service Foundation Trust, Wirral; Richard Cowan, Christie Hospital, University of Manchester, Manchester; John Anderson, Sheffield Teaching Hospitals, National Health Service Foundation Trust, Sheffield; John Hetherington, Castle Hill Hospital, Hull; Salah El-Sharkawi, South West Wales Cancer Centre, Swansea; P.D. John Hardman, The James Cook University Hospital, Middlesbrough, United Kingdom; Wendy R. Parulekar, Andrea Hiltz, and Bingshu E. Chen, NCIC Clinical Trials Group, Queen's University; Michael Brundage, Cancer Centre of Southeastern Ontario, Kingston; Mary Gospodarowicz and Padraig Warde, University of Toronto, Princess Margaret Cancer Centre; Charles Hayter, University of Toronto, Carlo Fidani Peel Regional Cancer Center, Toronto, Ontario; Edmund C. Kostashuk, Fraser Valley Cancer Centre, Surrey; Michael McKenzie, Vancouver Cancer Centre, Vancouver, British Columbia; Jinka Sathya, Memorial University of Newfoundland, St Johns, Newfoundland and Labrador, Canada; Luis Souhami, McGill University, Montreal, Quebec, Canada; and Gregory Swanson, University of Texas Health Science Center, San Antonio, TX.

## Abstract

**Purpose:**

We have previously reported that radiotherapy (RT) added to androgen-deprivation therapy (ADT) improves survival in men with locally advanced prostate cancer. Here, we report the prespecified final analysis of this randomized trial.

**Patients and Methods:**

NCIC Clinical Trials Group PR.3/Medical Research Council PR07/Intergroup T94-0110 was a randomized controlled trial of patients with locally advanced prostate cancer. Patients with T3-4, N0/Nx, M0 prostate cancer or T1-2 disease with either prostate-specific antigen (PSA) of more than 40 μg/L or PSA of 20 to 40 μg/L plus Gleason score of 8 to 10 were randomly assigned to lifelong ADT alone or to ADT+RT. The RT dose was 64 to 69 Gy in 35 to 39 fractions to the prostate and pelvis or prostate alone. Overall survival was compared using a log-rank test stratified for prespecified variables.

**Results:**

One thousand two hundred five patients were randomly assigned between 1995 and 2005, 602 to ADT alone and 603 to ADT+RT. At a median follow-up time of 8 years, 465 patients had died, including 199 patients from prostate cancer. Overall survival was significantly improved in the patients allocated to ADT+RT (hazard ratio [HR], 0.70; 95% CI, 0.57 to 0.85; *P* < .001). Deaths from prostate cancer were significantly reduced by the addition of RT to ADT (HR, 0.46; 95% CI, 0.34 to 0.61; *P* < .001). Patients on ADT+RT reported a higher frequency of adverse events related to bowel toxicity, but only two of 589 patients had grade 3 or greater diarrhea at 24 months after RT.

**Conclusion:**

This analysis demonstrates that the previously reported benefit in survival is maintained at a median follow-up of 8 years and firmly establishes the role of RT in the treatment of men with locally advanced prostate cancer.

## INTRODUCTION

Prostate cancer is the most common cancer diagnosed in men in the Western Hemisphere, with approximately 899,000 patients diagnosed and 258,000 deaths worldwide in 2008.^1^ Patients with locally advanced disease, defined as stage categories T3-4, N0, and M0, are still prevalent in regions where the use of prostate-specific antigen (PSA) screening is not widespread.^2^

Previous uncertainties about the roles of radiotherapy (RT) and androgen-deprivation therapy (ADT)^3,4^ have been greatly clarified after the publication of randomized trials demonstrating the benefits of ADT added to RT and the benefits of RT added to ADT.^5–7^ Three reported randomized trials compared ADT alone with to ADT plus RT. The present trial was the largest of these and was developed by the NCIC Clinical Trials Group in collaboration with the Medical Research Council and the National Cancer Institute US Cancer Therapy Evaluation Program. The interim analysis of this intergroup trial has been reported previously^8^ and showed a significant overall survival (OS) improvement for patients treated with ADT+RT (hazard ratio [HR], 0.77; 95% CI, 0.61 to 0.98; *P* = .033) and improvement in disease-specific survival (DSS). The final, preplanned analysis presented here reports on the longer-term survival outcomes and toxicity. Quality-of-life analyses are reported by Brundage et al.^8a^

## PATIENTS AND METHODS

The study design has been previously described in detail.^8^ Patients were randomly assigned to ADT alone or to ADT+RT. Eligible patients had locally advanced disease, initially defined as T3-4, N0, M0. In 1999, the entry criteria were broadened to include patients with localized (T1-2) but high-risk disease, defined either as a PSA of more than 40 μg/L or PSA of 20 to 40 μg/L plus a Gleason score of 8 to 10. Pelvic node imaging was not mandatory unless only the prostate was to be irradiated, rather than the whole pelvis. Surgical lymph node staging before random assignment was permitted and had to be negative for nodal disease. No previous therapy for prostate cancer was allowed, but random assignment was permitted within a 12-week window after starting first-line ADT.

The primary objective was to determine whether the addition of RT to ADT prolonged OS, defined as time from random assignment to death from any cause or censoring at last follow-up. Secondary end points were time to progression (TTP), DSS, quality of life, toxicity, and symptomatic local control (defined as surgical interventions for symptomatic local disease).

Disease progression was defined as the first of any of the following events: biochemical progression, local progression, development of metastatic disease, or death from prostate cancer. For the per-protocol analysis, biochemical progression was defined by two consecutive PSA readings of more than 10 ng/mL in patients whose PSA nadir was ≤ 4 ng/mL. In patients whose PSA nadir was greater than 4 ng/mL, biochemical progression was defined as a PSA level of more than 10 ng/mL and 20% above the baseline reading. In addition to this prespecified definition, we analyzed biochemical progression according to the American Society for Radiation Oncology Phoenix criteria.^9^ Local progression was defined either after histologic confirmation or after the development of ureteric obstruction. Distant progression was defined by imaging.

Patients were randomly assigned using a straight minimization strategy,^10^ stratified by center, initial PSA level (< 20 *v* 20 to 50 *v* > 50 μg/L), choice of hormonal therapy (luteinizing hormone–releasing hormone [LHRH] agonist *v* orchiectomy), method of lymph node staging (clinical *v* surgical *v* not done), Gleason score (< 8 *v* 8 to 10), and prior hormonal therapy.

ADT consisted of either bilateral orchiectomy or LHRH agonists (plus 2 weeks of oral antiandrogen to cover flare), according to patient and physician preference, and was continued for life. RT was to start within 8 weeks after random assignment and was given to the whole pelvis, to a dose of 45 Gy in 25 fractions, followed by a further 20 to 24 Gy to the prostate alone in 10 to 12 fractions. The total treatment time was 7 to 7.5 weeks. If the treating physician felt that a patient was unsuitable for whole pelvic RT or if histologically negative lymph nodes had been demonstrated, RT to the prostate alone, to a dose of 64 to 69 Gy in 35 to 39 fractions, was permitted. Pelvic RT was delivered using a four-field box technique to cover the prostate, seminal vesicles, and internal and external iliac lymph nodes. The prostate volume encompassed the prostate and periprostatic tissues with a margin at the investigating physician's discretion. Patients in Canada underwent real-time review of their RT implementation. The dose distributions, treatment prescription sheets and simulator films for Canadian patients were reviewed by one author (C.H.) before treatment or at latest within 3 days of start of treatment, and recommendations for change, if necessary, were faxed back to the prescribing physician within 24 hours. After the completion of RT, copies of all completed prescription sheets were sent for review to ascertain that the treatment was delivered according to protocol. The dose was specified at the intersection of the beam axes, according to the guidelines of the International Commission on Radiation Units.^11^ The study received the required local and national ethics committee approvals; all patients signed an informed consent document.

The original study design mandated accrual of 650 patients to the trial, based on an assumed 10-year survival of 35% for patients treated with ADT only, to detect a 10% improvement in 10-year survival (HR of 0.76, 80% power using a one-sided 5% level test). In September 2002, after 688 patients had been recruited, the protocol was amended because of a low event rate. The revised statistical parameters assumed a 57% survival rate at 10 years in the control arm, 80% power, and an overall 2.5% level one-sided test to detect the same HR of 0.76, which would translate to an absolute 8.4% increase in 10-year survival. This required a total of 421 deaths for the final analysis, taking two planned interim analyses into account. On the basis of the type I error spending function as proposed by DeMets and Lan,^12^ the prespecified significance level for final analysis was *P* = .035 (two-sided) to maintain the overall significant level of two-sided *P* = .05. According to these specifications, the estimated sample size for the study was 1,200.

OS was determined using the Kaplan-Meier product-limit method^13^ and compared using a log-rank test stratified for initial PSA level, choice of hormonal therapy, method of lymph node staging, Gleason score, and prior hormonal therapy. Two Cox model analyses were used. First, HRs and CIs were estimated (Cox model 1).^14^ The DSS rates were estimated by cumulative incidence. The analyses were performed with the use of SAS software, version 9.2 (SAS Institute, Cary, NC). Second, a Cox proportional hazards model was used to assess the treatment effect while adjusting for known important prognostic factors in addition to the prespecified stratification factors (Cox model 2). These were region (North America *v* rest of world), initial PSA level (< 20 *v* 20 to 50 *v* > 50 μg/L *v* missing), Gleason score (< 8 *v* 8 to 10 *v* missing), prior hormone therapy (excluding orchiectomy; yes *v* no), choice of hormonal therapy (orchiectomy *v* LHRH agonist), method of lymph node staging (clinical [no computed tomography] *v* radiologic [computed tomography negative] *v* surgical dissection *v* not done or missing), age (< *v* ≥ 65 years), and orchiectomy versus LHRH. TTP was defined as the time from random assignment to the date of the first documented disease progression or death from prostate cancer.

Toxicity was measured using the NCIC Clinical Trials Group Expanded Common Toxicity Criteria. Information on quality of life is presented by Brundage et al.^8a^

## RESULTS

**Fig 1.**
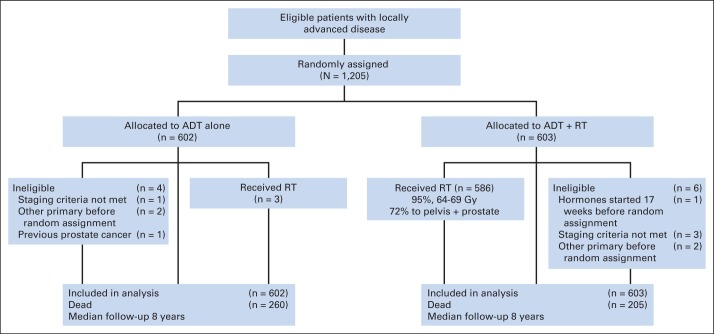
CONSORT diagram. ADT, androgen-deprivation therapy; RT, radiotherapy.

Between 1995 and 2005, 1,205 patients were randomly assigned (Fig 1). Trial participants were well matched in terms of their baseline characteristics (Table 1). Their median age was 70 years. Eighty-seven percent of patients had locally advanced (T3-4) disease, 63% of patients had a PSA of more than 20 μg/L, and 18% had a Gleason score of more than 8. The database contained data up to and including December 31, 2010, and included 465 reported deaths. The median follow-up time was 8 years (range, 0 to 15.2 years). Ninety-four percent of patients included in the analysis had data available in the 2 years preceding the clinical cutoff date.

**Table 1.** Baseline Demographics and Clinical Characteristics

Characteristic	ADT (n = 602)		ADT+RT (n = 603)		Total (N = 1,205)	
	No. of Patients	%	No. of Patients	%	No. of Patients	%
Age, years						
< 65	134	22.3	132	21.9	266	22.1
≥ 65	468	77.7	471	78.1	939	77.9
Median	69.7		69.7		69.7	
Range	50-79.8		46.3-80.3		46.3-80.3	
ECOG performance status						
0	474	78.7	469	77.8	943	78.3
1	119	19.8	126	20.9	245	20.3
2	9	1.5	8	1.3	17	1.4
Lymph node staging						
Clinical	427	70.9	422	70.0	849	70.5
Radiologic	50	8.3	53	8.8	103	8.5
Surgical	12	2.0	17	2.8	29	2.4
Not done	113	18.8	111	18.4	224	18.6
T stage						
Missing			1	0.2	1	0.1
T2	76	12.6	71	11.8	147	12.2
T3	499	82.9	501	83.1	1000	83.0
T4	27	4.5	30	5.0	57	4.7
Rectal exam						
Done	586	97.3	588	97.5	1174	97.4
Not done	16	2.7	15	2.5	31	2.6
Results of rectal examination						
Abnormal	161	26.7	162	26.9	323	26.8
Normal	19	3.2	19	3.2	38	3.2
Unknown/missing	422	70.1	422	70.0	844	70.0
Region of patients						
Northern America	180	29.9	181	30.0	361	30.0
MRC	422	70.1	422	70.0	844	70.0
Initial PSA level, μg/L						
< 20	223	37.0	216	35.8	439	36.4
20-50	229	38.0	231	38.3	460	38.2
> 50	150	24.9	156	25.9	306	25.4
Gleason score						
Missing	6	1.0	3	0.5	9	0.7
< 8	380	63.1	381	63.2	761	63.2
8-10	216	35.9	219	36.3	435	36.1
ADT before random assignment						
No	314	52.2	315	52.2	629	52.2
Yes	288	47.8	288	47.8	576	47.8
Choice of hormonal therapy						
LHRH	562	93.4	562	93.2	1124	93.3
Bilateral orchiectomy	40	6.6	41	6.8	81	6.7

Abbreviations: ADT, androgen-deprivation therapy; ECOG, Eastern Cooperative Oncology Group; LHRH, luteinizing hormone–releasing hormone; MRC, Medical Research Council; PSA, prostate-specific antigen; RT, radiotherapy.

Of the 603 patients randomly assigned to ADT+RT, 586 (97%) received RT, and 13 did not receive RT; in four patients, it was unknown whether or not RT was received. Of the 586 patients known to have received RT, 43 received doses less than 65 Gy, and 10 received doses greater than 69 Gy. Thus, 88% of the patients allocated to the ADT+RT arm received doses between 65 and 69 Gy. Nine (1%) of 602 patients randomly assigned to ADT alone received RT, as defined by irradiation to the pelvis of more than 50 Gy within 1 year of random assignment and without evidence of disease progression. LHRH agonists were used in 1,105 patients (92%), and bilateral orchiectomy was performed in 93 patients (8%), with no evidence of differences in proportions between the two arms.

### OS

**Fig 2.**
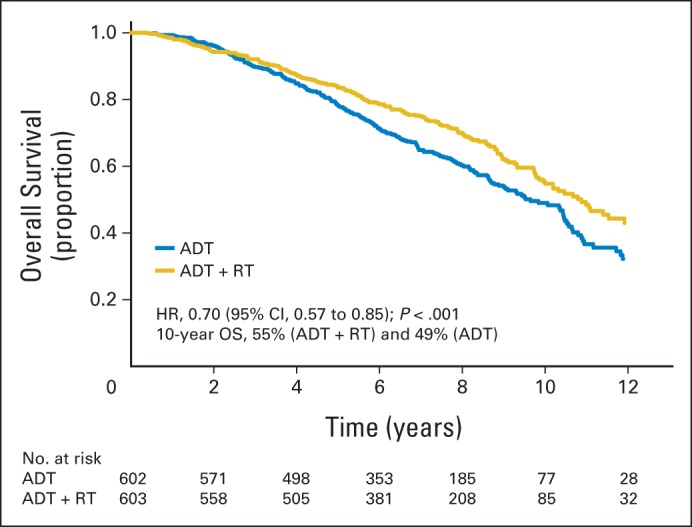
Overall survival (OS). ADT, androgen-deprivation therapy; HR, hazard ratio; RT, radiotherapy.

There were 260 deaths reported in patients treated with ADT alone and 205 deaths in patients treated with ADT+RT. The addition of RT led to a 30% reduction in the risk of death (HR, 0.70, based on Cox model 1; 95% CI, 0.57 to 0.85; *P* < .001; Fig 2). The median OS time was 9.7 years (95% CI, 8.8 to 10.5 years) for patients on the ADT-alone arm, whereas it was 10.9 years (95% CI, 10.0 to 12.8 years) for patients on the ADT+RT arm. The 10-year OS rate was 49% (95% CI, 44% to 54%) for patients on the ADT arm, whereas it was 55% (95% CI, 49% to 60%) for patients on the ADT+RT arm. A multivariable Cox model confirmed the effect of treatment, independent from other variables, with a *P* = .0011 in favor of the ADT+RT arm. The adjusted HR of ADT+RT versus ADT alone was 0.74 (95% CI, 0.61 to 0.87, based on Cox model 2). Both PSA level (> 50 *v* < 20 μg/L) and Gleason score (8 to 10 *v* < 8) were significant prognostic factors for OS.

### DSS

**Fig 3.**
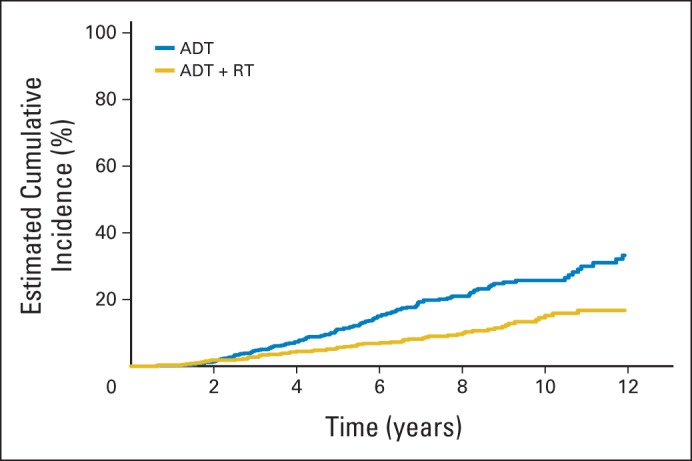
Deaths from prostate cancer. ADT, androgen-deprivation therapy; RT, radiotherapy.

Analysis of DSS indicated an excess of deaths caused by prostate cancer in patients treated with ADT alone (Table 2). A competing risks analysis indicated a significant reduction in the risk of death from prostate cancer in patients treated with ADT+RT (HR, 0.46; 95% CI, 0.34 to 0.61; *P* < .001; Fig 3). There was no evidence of any differences in deaths from other causes (*P* = .58; Table 2). Sensitivity analyses were performed to test the impact of potential inaccuracy in investigator assignment of cause of death. In each case, the reduction in risks of death from prostate cancer in RT-treated patients was confirmed, with *P* < .001

**Table 2.** Causes of Death

Cause of Death	ADT (n = 260)		ADT+RT (n = 205)		Total (n = 465)	
	No. of Patients	%	No. of Patients	%	No. of Patients	%
Prostate cancer	134	52	65	32	199	43
Cardiac/stroke	37	14	33	16	70	15
Other cancer	31	12	44	17	75	16
Pneumonia	11	4	11	9	22	5
Other	31	12	34	21	65	14
Unknown	16	6	18	5	34	7
Alive	342		398		740	

Abbreviations: ADT, androgen-deprivation therapy; RT, radiotherapy.

### Nonfatal End Points

#### Disease progression.

**Fig 4.**
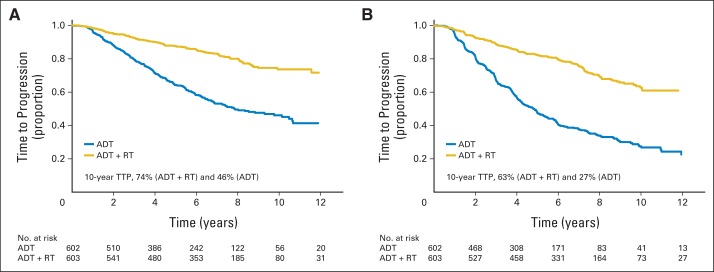
Time to disease progression (TTP; A) using prespecified definition of biochemical progression and (B) using American Society for Radiation Oncology Phoenix definition of biochemical progression. ADT, androgen-deprivation therapy; RT, radiotherapy.

Using the prespecified definition of biochemical progression, the 10-year disease progression–free rate was 46% (95% CI, 41% to 51%) for patients on the ADT-alone arm and 74% (95% CI, 68% to 78%) for patients on the ADT+RT arm. The HR for TTP on the ADT+RT arm versus the ADT-alone arm was 0.31 (95% CI, 0.25 to 0.39; Fig 4A).

Using the American Society for Radiation Oncology Phoenix definition of biochemical progression, the 10-year biochemical progression-free rate was 27% (95% CI, 23% to 32%) for patients on the ADT-alone arm, whereas it was 63% (95% CI, 57% to 68%) for patients on the ADT+RT arm. The HR for TTP on the ADT+RT arm versus the ADT arm was 0.31 (95% CI, 0.27 to 0.37).

#### Toxicity.

The five most frequent grade 3 or higher treatment-related toxicities were impotence/libido (29% for ADT alone and 33% for ADT+RT; *P* = .17), hot flushes (8% for ADT alone and 5% for ADT+RT; *P* = .10), urinary frequency (4% for ADT alone and 7% for ADT+RT; *P* = .13), ischemia (3% for ADT alone and 5% for ADT+RT; *P* = .09), and hypertension (3% for ADT alone and 4% for ADT+RT; *P* = .54). There was no evidence of a difference in reported cardiovascular toxicities between the two arms. Bowel-related adverse events were more frequent in the ADT+RT arm compared with the ADT arm; most were grade 1 and 2. The rate of bowel adverse events greater than grade 3 at 24 months was negligible (two of 589 assessable patients in the ADT+RT arm (Table 3).

**Table 3.** Bowel-Related Adverse Events at 24 Months

Toxicity	No. of Patients								
	ADT (n = 606)			ADT+RT (n = 589)			Total		
	Any	Grade 1-2	Grade 3-5	Any	Grade 1-2	Grade 3-5	Any	Grade 1-2	Grade 3-5
Diarrhea	87	14	0	223	32	2	310	46	2
Flatulence	20	1	1	37	7	0	57	8	1
Bleeding	50	5	0	133	41	0	183	46	0
Pain	62	12	1	79	10	0	141	22	1
Proctitis	43	4	0	119	19	0	162	23	0

Abbreviations: ADT, androgen-deprivation therapy; RT, radiotherapy.

### Effect of RT Field

Using data prospectively reported by investigators, we performed exploratory post hoc analyses (uncorrected for multiple comparisons) to examine the effects of RT field among patients allocated to the ADT+RT arm, stratified by region, PSA, Gleason score, choice of hormone therapy, lymph node staging, and prior hormone therapy. Patients planned for pelvic irradiation (n = 420, 72%) showed a trend toward improved OS compared with prostate-only RT (n = 166, 28%; HR, 0.70; 95% CI, 0.45 to 1.09; *P* = .12). The other efficacy analyses were as follows: for TTP, the HR was 0.77 (95% CI, 0.44 to 1.34; stratified log-rank *P* = .35), and for DSS, the HR was 0.53 (95% CI, 0.25 to 1.13; *P* = .098).

## DISCUSSION

These results confirm the previous, interim findings from our randomized controlled trial,^8^ with RT improving both overall and prostate cancer–specific outcomes (DSS, TTP) when added to ADT in men with locally advanced disease. Furthermore, this was achieved without major detriment in terms of long-term toxicity. Our results concur with those from other trials in similar groups of patients. The Swedish Prostate Cancer Group (SPCG) study SPCG-7^15^ showed a similar reduction in overall mortality (HR, 0.68) and disease-specific mortality (HR, 0.44) for the addition of RT to flutamide. A study (ClinicalTrials.gov identifier: NCT01122121) by the French collaborative group in 264 patients with T3-4 disease reported a significant improvement in progression-free survival with the addition of RT to ADT.^16^ Median OS times had not been reached in that study, and no improvement in OS was detected; however, this trial was relatively small and was powered only to detect a difference in 5-year progression-free survival and not a difference in OS.

Our results are noteworthy for a number of reasons. First, they indicate that the benefits seen for RT in the context of ADT with flutamide (in the SPCG-7 study) are also seen with LHRH agonist therapy or surgical castration. Second, our patient population represents a higher risk group than in SPCG-7; the Swedish group had pathologic confirmation of N0 status if the PSA was greater than 11 ng/mL (2% of our study), 20% had T1-2 disease (10% in our study), 60% had a PSA of less than 20 ng/mL (37% of our study), and the maximum allowed PSA level was 70 ng/mL (unlimited in our study), although 75% of patients had WHO grade 1 or 2 tumors, (81% in our study had Gleason score < 7). Third, the median follow-up in our study was 8 years compared with 7.6 years in the SPCG-7 study.

The RT technique used in our trial reflects the prevailing treatment philosophies of the time. The study predated outcome data from randomized trials of dose-escalated RT, and the RT doses used here are modest by modern standards.^17–19^ Whether dose escalation in this setting might achieve superior outcomes even to those reported here is a matter for speculation. Data from dose-escalation studies suggest that biochemical control might be as much as two-fold better for RT doses greater than 70 Gy,^20^ and there is no evidence that patients with T3-4 disease do not realize such benefits from dose escalation.^17,20^ A recent study using ADT plus dose-escalated RT (80 Gy in 40 fractions or equivalent) in 168 patients with high-risk prostate cancer reported a biochemical progression-free rate of 79% at 5 years.^21^

Another unanswered question relates to the optimum field of RT. This question is still unresolved despite several randomized trials.^22–24^ Our nonrandomized, post hoc exploratory subgroup analysis indicates a trend toward improved outcome with larger field size, a finding that requires confirmation in a rigorously conducted phase III randomized controlled trial. These results must be interpreted with caution because of biases inherent with selection of RT field in our study (ie, the influence of comorbidities or other patient factors that may confound the efficacy analyses) and the potential for more toxicity associated with a wider field of treatment delivery. Ongoing trials, such as the United Kingdom PIVOTAL trial (ClinicalTrials.gov identifier: NCT01685190; A Randomised Phase II Trial of Prostate and Pelvic Versus Prostate Alone Radiotherapy Treatment Volumes Using High-Dose IMRT for Locally Advanced Prostate Cancer), currently a randomized phase II study, or the Radiation Therapy Oncology Group 0924 phase III trial (ClinicalTrials.gov identifier: NCT01368588), will establish whether the addition of high-dose nodal irradiation using intensity-modulated RT improves outcomes compared with prostate-only RT in patients with N0M0 disease. Some patients, staged clinically as N0, would be found to have pathologic evidence of lymph node metastases if subjected to lymph node dissection, and it might be argued that our findings for pelvic RT suggest a benefit for such treatment in patients with clinical or pathologic node-positive disease. Alternatively, it is possible that the larger fields used for pelvic irradiation might actually have achieved better coverage of the prostate itself, compared with prostate-only fields as used in the pre–intensity-modulated RT era, and the trends seen could reflect this. However, our data on local control in the prostate do not permit further exploration of this possibility here.

As would be expected, our toxicity data indicate a detectable, although modest, impact of RT as administered in this trial. It is noteworthy that the grade ≥ 3 toxicity that we detected was short term only, and we would suggest that the toxicity of RT should not be regarded as a barrier to its routine use in this patient population.

In this trial, the intended duration of ADT was lifelong. Data have emerged elsewhere on the long-term toxicities of ADT,^25–27^ and we are unable to provide data regarding optimal duration because of the symmetry and long-term nature of ADT in both treatment arms of our study. For high-risk disease, data from clinical studies support longer (> 2 years) rather than shorter durations (≤ 6 months).^5^

This trial underlines the benefits of achieving local control with RT in locally advanced prostate cancer. Surgery might also be an effective means of achieving local disease control,^2,28^ but given the Level I evidence presented here and by others,^15^ alternatives to ADT+RT should only be administered in the context of a prospective randomized controlled trial.

Recent data suggest that some men with T3-4 disease are still being managed with ADT alone.^29,30^ Although there are undoubtedly patients for whom RT or indeed any curative therapy would be inappropriate because of age, comorbidity, or other factors, we conclude that patients with clinically node-negative, locally advanced prostate cancer who are suitable for additional RT should be offered that option, an opinion shared by European^31^ and North American^32^ guidelines.

## Supplementary Material

Protocol

## Glossary Terms

Gleason score: pathologic description of prostate cancer grade on the basis of the degree of abnormality in the glandular architecture. Gleason patterns 3, 4, and 5 denote low, intermediate, and high levels of histologic abnormality and tumor aggressiveness, respectively. The score assigns primary and secondary numbers on the basis of the most common and second most common patterns identified.
intensity-modulated radiation therapy: radiation treatment using beams with nonuniform fluence profiles that shape the dose distribution in the target volume and adjacent normal structures. Beam modulation is typically achieved via multileaf collimators or custom-milled compensators to achieve the appropriate fluence profiles calculated by inverse optimization algorithms. The radiation beam is divided into beamlets of varying intensity such that the sum from multiple beams via inverse planning results in improved tumor targeting and normal tissue sparing. A technique of radiation therapy delivery in which the intensity of each beamlet of radiation coming from a specific angle can be adjusted to provide a desired dose distribution when the doses delivered from all beamlets are added from a single angle and from all dose delivery angles. An advanced type of high-precision radiotherapy, which aims to improve the coverage of the radiotherapy target and/or minimize radiation dose to surrounding normal tissue.
overall survival: the duration between random assignment and death.

## Appendix

The NCIC Clinical Trials Group (CTG) PR.3/Medical Research Council (MRC) UK PR07 investigators were as follows: *Central Office staff:* Canada—K. James, M. Bacon, A. LeMaitre, K. Ding (NCIC CTG, Kingston, Ontario, Canada). *MRC Clinical Trials Unit staff: UK*—M.R. Sydes, S.L. Naylor, N. Kelk, J. Latham, J. Nuttall, K. Sanders (trial managers), M.K.B. Parmar, M.R. Sydes, R.C. Morgan, G. Jovic (statisticians; MRC, London, United Kingdom). *Collaborators: Canada*—P. Joseph, D. Wilke (Queen Elizabeth II Health Sciences Centre, Halifax, Nova Scotia); M. Duclos, S. Faria, C. Freeman, L. Souhami (McGill University, Montreal, Quebec); J.P. Bahary, J.P. Guay (CHUM Hopital Notre-Dame, Montreal, Quebec); L. Eapen (Ottawa Health Research Institute General Division, Ottawa, Ontario); C. Hayter (Cancer Centre of South-Eastern Ontario at Kingston, Kingston, Ontario); T. Corbett, H. Lukka, M. Patel (Juravinski Centre at Hamilton Health Sciences, Hamilton, Ontario); L. Klotz (Odette Cancer Centre, Toronto, Ontario); A. Bayley, R. Bristow, C. Catton, J. Crook, M. McLean, M. Milosevic (University Health Network–Princess Margaret Hospital, Toronto, Ontario); J. Radwan, G. Rodrigues (London Regional Cancer Program, London, Ontario); C. Springer (Windsor Regional Cancer Centre, Windsor, Ontario); B. Lada, H. Prichard, R. Samant (Northeastern Ontario Cancer Centre, Sudbury, Ontario); H. Dhaliwal, S. Gulavita (Thunder Bay Regional Health Science Centre, Thunder Bay, Ontario); R. Halperin, D. McGowan, M. Parliament (Cross Cancer Institute, Edmonton, Alberta). *United Kingdom*—P. Kirkbride, J. Anderson (Weston Park and Royal Hallamshire Hospitals, Sheffield); M. Mason, J. Barber (Velindre Hospital, Cardiff); J. Heatherington, C. Bevis (Castle Hill Hospital, Hull); I. Pedley, R. McMenimen, T. Roberts (Newcastle General Hospital, Newcastle-upon-Tyne); A. El Sharkawi (Singleton Hospital, Swansea); J. Hardman, H. Van der Voet (The James Cook University Hospital, Middlesborough); D. Dearnaley, R. Huddart, A. Horwich, R. Eeles (Royal Marsden Hospitals, Sutton); I. Syndikus, J. Littler, D. Errington, A. Shenoy (Clatterbridge Centre for Oncology, Bebington); D. Sheehan, R. Srinivasan (Royal Devon and Exeter, Exeter); A. Lydon (Torbay Hospital, Torbay); S. Sundar, M. Sokal (Nottingham City Hospital, Nottingham); C. Loughrey, S. Stranex (Belfast City and Belvoir Park Hospitals, Belfast); P. Rogers, A. Folkes (Royal Berkshire and Battle Hospitals, Berkshire); A. Stockdale, C. Irwin (Walsgrave Hospital, Coventry); R. Owen, P. Jenkins, A. Ritchie (Gloucestershire Hospitals, Cheltenham); R. Cowan, J. Wylie (Christie Hospital, Manchester); A. Al-Samarraie, S. Gollins, R. Russell (Glan Clwyd Hospital, Rhyl); N. Srihari, A. Cook, C. Beacock (Royal Shrewsbury Hospital, Shrewsbury); C. Parker, T. Porter (Yeovil District Hospital, Yeovil); P. Symmonds, M. Madden (Leicester Royal Infirmary, Leicester); S. Susnerwala (Blackpool Victoria Hospital, Blackpool); D. Bloomfield (Brighton Hospitals, Brighton); P. Whelan (St James's University Hospital, Leeds); D. Cole, E. Sugden (Churchill Hospital, Oxford); T. Sreenivasan (United Lincolnshire Trust, Lincoln); A. Harnett, T. Kumar, R. Mills (Norfolk and Norwich Hospital, Norwich); T. Sreenivasan, S. Dixit (Scunthorpe General Hospital, Scunthorpe); H. Newman (Royal United Hospital, Bath); C. Powell (Countess of Chester Hospital, Chester); H. Algurafi (Mayday Hospital, Croydon); S. Beesley (The Kent Cancer Centre, Maidstone); C. Elwell (Northampton Oncology Centre, Northampton); S. Sundaram, M. Murphy, P. Weston (Pinderfields Hospital, Wakefield); A. Al-Samarraie (Wrexham Maelor Hospital, Wrexham); J. Graham, S. Falk (Bristol Oncology Centre, Bristol); M. Russell (Beatson Oncology Centre, Glasgow); D. Bottomley, A. Kiltie (Cookridge Hospital, Leeds); A. Robinson, O. Koriech (Southend General Hospital, Southend); C. Woodward (West Suffolk Hospital, Bury St Edmunds); D. Wheatley, R. Ellis (Royal Cornwall Hospital, Truro); I. Syndikus (Warrington Hospital, Warrington); M. Stower, G. Urwin (York District Hospital, York); F. Bramble, J. Rundle, C. Carter (Bournemouth and Christchurch Hospitals); G. Sole (Hereford County Hospital, Hereford); C. Heath (Royal South Hants Hospital, Southampton); D. Bloomfield (Sussex Oncology Centre, Brighton); M. Pantelides (Royal Bolton Hospital, Bolton); H. Patterson (Addenbrooke's Hospital, Cambridge); S. Dixit (Diana Princess of Wales Hospital, Grimsby); D. Fermont, R. Shah (Mount Vernon Hospital, Northwood); C. Read (Royal Preston Hospital, Preston); A. El-Modir (Sandwell General Hospital, West Bromwich); S. Beesley (Conquest Hospital, St Leonards-on-Sea); D. Cole (The Great Western Hospital, Swindon); R. Wilson (Taunton and Somerset Hospital, Taunton); C. Humber (Warwick Hospital, Warwick); A. Samanci, B. Waymont (New Cross Hospital, Wolverhampton); D. Bissett (Aberdeen Royal Infirmary, Aberdeen); P. Chakraborti (Queen's Hospital, Burton-upon-Trent); F. Mckinna (Eastbourne Hospital, Eastbourne); I. Syndikus (Southport and Formby District General Hospital, Southport); J. Glaholm (Good Hope Hospital, Sutton Coldfield). *Russia*—O. Kariakine (MRRC RAMS, Obninsk). *New Zealand*—C. Jose (Auckland Hospital, Auckland). *South Africa*—A. Abratt (Groote Schuur Hospital, Cape Town).
