# Supplementary material for: Final Report of the Intergroup Randomized Study of Combined Androgen-Deprivation Therapy Plus Radiotherapy Versus Androgen-Deprivation Therapy Alone in Locally Advanced Prostate Cancer
Source: J Clin Oncol. 2015 Feb 17;33(19):2143–50. doi: 10.1200/JCO.2014.57.7510 (PMC4477786; doi:10.1200/JCO.2014.57.7510)
Supplement: Protocol [file supp_JCO.2014.57.7510_Protocol_JCO.2014.57.7510.pdf]

The following protocol information is provided solely to describe how the authors conducted the research underlying the published report associated with the following article:

Final Report of the Intergroup Randomized Study of Combined Androgen Deprivation Therapy Plus Radiotherapy Versus Androgen Deprivation Therapy Alone in Locally Advanced Prostate Cancer

Mason, et al

DOI: 10.1200/JCO.2014.57.7510

The information provided may not reflect the complete protocol or any previous amendments or modifications. As described in the Information for Contributors (http://jco.ascopubs.org/site/ifc/protocol.xhtml) only specific elements of the most recent version of the protocol are requested by *JCO*. The protocol information is not intended to replace good clinical judgment in selecting appropriate therapy and in determining drug doses, schedules, and dose modifications. The treating physician or other health care provider is responsible for determining the best treatment for the patient. ASCO and *JCO* assume no responsibility for any injury or damage to persons or property arising out of the use of these protocol materials or due to any errors or omissions. Individuals seeking additional information about the protocol are encouraged to consult with the corresponding author directly.

PROTOCOL DATE: 95-JAN-23
NCIC CTG TRIAL: PR.3

REVISED: 95-JUN-13; AMENDED: 96-APR-29; AMENDED/REVISED: 96-JUL-25
REVISED: 96-SEP-05; AMENDED: 97-AUG-01; AMENDED: 99-FEB-19
REVISED: 99-OCT-14; REVISED: 01-OCT-29, AMENDED: 03-FEB-05
AMENDMENT: 2004-JAN-23; AMENDED: 2008-MAY-08; AMENDED 2009-MAR-18

NCIC CLINICAL TRIALS GROUP

NCIC CTG: PR.3 (INT: T94-0110)

SWOG: JPR.3, MRC-UK: PR07

INTERGROUP (NCIC CTG, CUOG, SWOG, MRC-UK) PHASE III RANDOMIZED TRIAL COMPARING TOTAL ANDROGEN BLOCKADE VERSUS TOTAL ANDROGEN BLOCKADE PLUS PELVIC IRRADIATION IN CLINICAL ADENOCARCINOMA OF THE PROSTATE

NCIC Clinical Trials Group (NCIC CTG)

Study Chair:	Dr. Padraig Warde,<br>Princess Margaret Hospital, Toronto, Ontario<br>416-924-0671 Ext. 5387; Fax: 416-926-6586
Study Co-chair:	Dr. Edmund Kostashuk
Trial Committee:	Dr. David Bell, Dr. David McGowan
GU Committee Chair:	Dr. Mary Gospodarowicz
Quality of Life Co-chair:	Dr. Andrea Bezjak
Radiotherapy Reviewer:	Dr. Charles Hayter
Physician Coordinator:	Dr. Wendy Parulekar
Biostatistician:	Dr. Keyue Ding
Study Coordinator:	Ms. Andrea Hiltz

	Southwest Oncology Group	MRC-UK
Study Co-chair:	Dr. Gregory P. Swanson<br>509-455-5930<br>Fax: 509-455-4023	Prof. Malcolm Mason<br>+44 (0) 1222 615 888<br>Fax: +44 (0) 1222 529 625
Trial Committee:		
GU Committee Chair:	Dr. E. David Crawford	
Prostate Committee Chair:	Dr. Ian Thompson	Mr. Leslie Moffat
Statistician:	Dr. Cathy Tangen	Mr. Matthew Sydes
Data Coordinator:	Kathy Bingham	Mr. Tim Smith
Protocol Editor:	Jennifer Scott	

(for NCI CTEP Use ONLY: VERSION DATE: 2009-MAR-18; AMENDMENT DATE: 2009-MAR-18)

TREATMENT SCHEMA

Patients with stage T3 or T4, N0 or NX, M0 or T2 PSA > 40 µg/L or T2 PSA > 20 µg/L and Gleason ≥ 8 adenocarcinoma of the prostate who have not had a radical prostatectomy.

Stratification:

Centre
Initial PSA level
Choice of hormonal therapy (orchiectomy or LHRH agonist)
Method of node-staging (clinical or radiological or surgical)
Gleason score
Prior hormone therapy (excluding orchiectomy)

RANDOMIZE

ARM A Antiandrogen (optional with orchiectomy)
- Flutamide 250 mg po TID
- or Nilutamide 100 mg po TID x 1 mo; then 150 mg po QD
- or Bicalutamide 50 mg po QD

PLUS (patient's choice)
- Bilateral orchiectomy
- or LHRH agonist
  - Goserelin 3.6 mg SC (abd) q28d
    - or 10.8 mg SC (abd) Q3mos
  - or Leuprolide 7.5 mg IM q28d (Leuprorelin 3.75 mg)
    - or 22.5 mg IM Q3mos (Leuprorelin 11.25 mg)
    - or 30 mg IM Q4mos
  - or Buserelin 6.3mg SC (abd) Q8wk
    - or 9.45mg SC (abd) Q12wk

ARM B Arm A treatment

PLUS Radical Radiation Therapy (65-69 Gy; 35-37 treatments)

→ Disease Progression → Death

Planned Sample: 1200

AMENDED: 96-APR-29; AMENDED: 97-AUG-01; AMENDED: 99-FEB-19

TABLE OF CONTENTS

TREATMENT SCHEMA 1

1.0 OBJECTIVES 1

2.0 BACKGROUND INFORMATION AND RATIONALE 2

3.0 BACKGROUND THERAPEUTIC INFORMATION 6
3.1 Anti-Androgens 6
3.2 LHRH Agonists 7

4.0 TRIAL DESIGN 10
4.1 Stratification 10
4.2 Randomization 11
4.3 Inclusion of Women and Minorities 11

5.0 STUDY POPULATION 12
5.1 Eligibility Criteria 12
5.2 Ineligibility Criteria 14

6.0 PRE-TREATMENT EVALUATION 15

7.0 ENTRY/RANDOMIZATION PROCEDURES 16
7.1 Entry Procedures 16
7.2 Stratification 17
7.3 Randomization: 17

8.0 TREATMENT PLAN 18
8.1 Hormonal Treatment Plan 18
8.2 Radiation Therapy (ARM B ONLY) 19
8.3 Concomitant Therapy 23

9.0 EVALUATION DURING STUDY 24

10.0 CRITERIA FOR MEASUREMENT OF STUDY ENDPOINTS 25
10.1 Overall Survival 25
10.2 Disease Specific Survival 25
10.3 Evidence of Disease Progression 25
10.4 Dating of Progression 25
10.5 Other Endpoints 26
10.6 Management Following Progression 26

11.0 ADVERSE EVENT REPORTING 27

12.0 PROTOCOL TREATMENT DISCONTINUATION 29
12.1 Criteria for Discontinuing Protocol Treatment 29

13.0 CENTRAL REVIEW PROCEDURES 30
13.1 Local Pathology Review 30
13.2 Central Radiotherapy Review 30

14.0 STATISTICAL CONSIDERATIONS 31
14.1 Objectives and Study Design 31
14.2 Study Endpoints and Analysis 31
14.3 Sample Size and Duration of Study 31
14.4 Sample Size for Quality of Life 32
14.5 Safety Monitoring 32
14.6 Interim Analysis 32

15.0 PUBLICATION POLICY 34
15.1 Authorship of Papers, Meeting Abstracts, Etc. 34
15.2 Responsibility for Publication 34
15.3 Submission of Material for Presentation or Publication 34

16.0 ETHICAL, REGULATORY AND ADMINISTRATIVE ISSUES 35
16.1 REB (Research Ethics Board) Approval for Protocols 35
16.2 Informed Consent 36
16.3 Centre Performance Monitoring 36
16.4 On-Site Monitoring 36
16.5 Case Report Forms 36

17.0 REFERENCES 37

18.0 SAMPLE CONSENT FORM 39

APPENDIX II - PERFORMANCE STATUS (ECOG) 48
APPENDIX III - STAGING CRITERIA 49
APPENDIX IV - DOCUMENTATION FOR STUDY 50
APPENDIX V - NCIC CTG EXPANDED COMMON TOXICITY CRITERIA 51
APPENDIX VI - LIST OF "CONTACTS" 58
APPENDIX VII - QUALITY OF LIFE ASSESSMENT 59

APPENDIX VIII - FRENCH CONSENT FORM 66

1.0 OBJECTIVES

To evaluate any possible benefit from the addition of external beam radiation therapy to the treatment of patients with locally advanced (T3 or T4, N0 or NX, M0 or T2, PSA >40 μg/L or T2, PSA >20 μg/L and Gleason ≥8) cancer of the prostate who have not had a radical prostatectomy and are receiving hormonal therapy in terms of:

1.1 The primary endpoint of overall survival

1.2 Secondary endpoints of:

1.2.1 Disease specific survival

1.2.2 Time to disease progression

1.2.3 Symptomatic local control as measured by the rates of surgical interventions necessary for symptomatic local disease (i.e. the combined incidences of trans-urethral resections, stent insertions, nephrostomies and colostomies).

1.2.4 Quality of life as measured by the QLQ-C30+3 and trial specific checklist (PR17) or FACT-P:

- describe quality of life of patients with prostate cancer treated with hormones from the time of diagnosis to death
- determine whether addition of prostate irradiation affects patients' quality of life
- assess responsiveness to change of two quality of life instruments (QLQ-C30 and FACT-P).

2.0 BACKGROUND INFORMATION AND RATIONALE

Adenocarcinoma of the prostate is the most common cancer and the second leading cause of cancer death among men in the United States. It is estimated that there will be 165,000 new cases of prostate cancer and 35,000 deaths in the United States in 1993 *(American Cancer Society, 1993)*. In Canada, prostate cancer is the second commonest malignancy in males and approximately 12,000 new cases will be diagnosed in 1993 *(Statistics Canada, 1993)*. Locally advanced disease (stage T3-4) accounts for 12 to 25% of cases. In the UK, locally advanced disease accounts for 20% of the approximately 10,000 new cases each year.

The optimal management of locally advanced prostate cancer is unclear. Surveys conducted by the American College of Surgeons compared patterns of practice in patients with T3 prostate cancer in 1974, 1983 and 1990 *(Mettlin, 1993)*. These studies demonstrated that over the past twenty years, the treatment approach has become more aggressive and a higher proportion of patients are now treated for cure. Approximately one quarter of patients with T3 tumours were treated with a curative approach in 1974, while over 50% were treated for cure in 1983. A large proportion of these patients were treated with radiation therapy (RT). More recently, an increased number of radical prostatectomies have been performed for patients with T3 disease, but this modality is still applied only in a minority of cases (10.4% in 1990). Despite these trends, many physicians treat patients with T3 prostate cancer with palliative intent using hormonal therapy alone, or with other simple measures. In continental Europe and the United Kingdom, most patients with organ-confined prostate cancer are routinely offered palliative therapy only *(Smith, 1990)*. A large survey undertaken in the UK showed that many patients receive hormone therapy or observation alone. Further, patients with T3 prostate cancer are included in phase III studies of hormonal therapy in the EORTC protocols.

Because the disease occurs in older men, many of whom have co-morbid conditions, it is frequently argued that no treatment is necessary. The natural history of untreated T3 prostate cancer is not clearly understood, and consequently it is difficult to assess the benefit of any form of therapy. The results of a prospective study of expectant management in patients with T3 disease conducted by Adolfsson showed that patients died at a higher rate than expected for an age matched population, but that not all the deaths were due to prostate cancer *(Adolfsson, 1993)*. The cancer specific and crude survival rates of patients in this study were similar to those in series where patients were treated with radiation therapy and radical prostatectomy. However, 22% of patients in this study developed bladder outlet obstruction necessitating surgical intervention and 30% developed distant metastases. While the benefit of treatment in this patient population has not been clearly established, it is clear that patients with untreated disease have significant disease related morbidity and mortality. When comparing results of observation in patients with T3 to those with T1 and T2 disease it was evident that, with prolonged follow-up, a much higher proportion of those with T3 disease died of prostate cancer.

The results of treatment of patients with T3 prostate cancer with radiation therapy alone are disappointing. Five year survival rates in large series lie between 50% to 70%, but at ten years these figures are down to 40% to 50% and the survival curves do not become parallel with those of an age matched population suggesting that these patients may not be cured *(Bagshaw, 1993; Babaian, 1990; Duncan, 1993)*. As with organ confined prostate cancer (T1, T2), local control rates obtained by RT in T3 prostate cancer are difficult to determine. Most studies report clinical local control rates as determined by recording the regression of palpable abnormalities on digital rectal examination (DRE) after external beam RT. Local recurrence after definitive radiotherapy is common, and is frequently associated with serious morbidity frequently requiring surgical intervention. In a series of 409 patients with T3 prostate cancer treated with external beam RT at Stanford University, the local control rates were 62% and 50% at 10 and 15 years respectively *(Bagshaw, 1993)*. Clinical local control in T3 tumours in 362 patients treated in a Princess Margaret Hospital (PMH) series was 57.5% at

10 years *(Duncan, 1993)*. In a series of 121 patients with T3 tumours treated with a combination of external beam RT and radioactive gold seed implantation reported by Holtzman, et al., 64 patients (53%) developed a local recurrence *(Holtzman, 1991)*. Forty-four of these patients had a bladder outlet obstruction, which necessitated transurethral resection and 16 of those became incontinent. Clearly, given these results, new approaches to the management of these patients are necessary.

It is difficult to assess the results of hormonal therapy in T3 prostate cancer, because most studies report these patients together with those with node positive or metastatic disease. A series of 115 untreated patients with clinical stage T3 cancer treated with complete androgen blockade has been reported by Dupont, et al *(Dupont, 1993)*. With an average follow-up of 3.9 years 28 patients had relapsed with a 10 year actuarial survival of 51%. The Medical Research Council in the United Kingdom has conducted a prospective randomized trial of orchiectomy alone, radiation therapy alone and combined orchiectomy and radiation therapy in patients with T2-T4 N0 M0 prostate cancer *(Fellows, 1992)*. This study was not completed, and the number of patients randomized (277 patients) was not sufficient to detect clinically relevant survival differences. However, there was no suggestion in this study that treatment with a hormonal approach alone compromised survival. There was also no difference in the need for further treatment for local progression (usually transurethral resection) between the three groups. Conversely, of patients referred for radiotherapy, a growing proportion have this treatment combines with hormone therapy. This has resulted from two recently published randomized trials which compared radiotherapy alone with radiotherapy plus hormone therapy. The Radiation Therapy Oncology Group (RTOG) 8531 study randomized 977 patients between these two options. Patients were included if they had T1-3 disease, with involved nodes (pelvic or para-aortic), T3 disease with no nodal or distant metastases, or were found to have positive margins after radical prostatectomy *(Pilepich, 1993)*. All involved areas were included in the radiotherapy fields, and hormone therapy was continued indefinitely: Local progression-free survival was significantly improved in patients receiving both modalities, as were metastasis-free survival and progression-free survival. There was no significant overall survival benefit, except when a subgroup analysis was performed, suggesting improved survival in patients with high grade tumours. The European Organization for Research and Treatment of Cancer (EORTC) randomized 415 patients with T1-2 (grade 3), or T3-4 N0 M0 patients to radiotherapy alone (two phases, whole pelvis plus prostatic boost), or radiotherapy plus hormone therapy for 3 years. Not only did this study also show a significant improvement in progression-free survival and local control, but also overall survival at 5 years. A third study, RTOG 8610, is awaited as a full publication *(Pilepich, 1998)*, but has explored a similar randomization in 471 patients with T3 and T4 disease. It has been reported as showing an improvement in local progression-free survival, and overall progression-free survival, but no significant effect on overall survival.

It is tempting to assume that these studies provide a rationale for the use of radiotherapy, supplemented by hormonal therapy in patients with locally advanced or poorer prognosis organ-confined cancers of the prostate. However, while they give added impetus to the use of hormone therapy in such patients, they cannot address the precise role of radiotherapy in such patients, as no study included a hormone therapy alone arm. Furthermore, a proportion of patients treated with radiotherapy will develop long-term side effects, occasionally severe, and these must be weighed against the possibility of any benefit.

The optimal hormonal therapy for prostate cancer is controversial. In the past, hormonal therapy usually involved bilateral orchiectomy alone, or with estrogen therapy, but in the last decade new hormonal agents were introduced, including pure antiandrogens and LHRH agonists *(Denis, 1993(a))*. Two prospective randomized studies have revealed a small, but significant, survival benefit in patients with metastatic disease when complete androgen blockade using an LHRH agonist and an antiandrogen was compared to standard management with either orchiectomy or LHRH monotherapy. *(Crawford, 1989; Denis, 1993 (b))* In a randomized study, although flutamide when added to orchiectomy produced statistically significantly more patients with PSA normalization, this was not associated with a better progression-free or overall survival. *(Eisenberger, 1997)* In commenting on this result, Raghaven *(Raghavan, 1997)* noted that a recent meta-analysis had shown that there was no difference between total androgen ablation and LHRH agonists or orchiectomy alone *(Prostate Cancer Trialists' Collaborative Group, 1995)* and that any benefit from the antiandrogen must be slight and perhaps entirely due to blocking the "flare" up of disease associated with the initiation of treatment. Randomized trials of complete androgen blockade versus monotherapy using a steroidal antiandrogen (e.g. Cyproterone Acetate) have not demonstrated a survival advantage. *(Robinson, 1988)*

In a recent survey of Canadian radiation oncologists and urologists carried out by the NCIC CTG Genito-urinary Disease Site Committee, 62% of radiation oncologists and 61% of urologists felt that the utility of radical RT in locally advanced prostate cancer had not been established *(Kostashuk, 1993)*. A study in the UK of 50 selected urologists and oncologists produced similar results. From responses in these surveys, it is clear that patients with locally advanced disease are treated with a wide variety of treatments, often with palliative intent. When faced with a patient with clinical stage T3/T4 prostate cancer, physicians have a major dilemma - whether to offer radical RT or not. Unfortunately, there is insufficient data in the literature to guide the clinician in this situation. Because it is not clear whether patients with T3, T4, prostate cancer can be cured, and there is controversy regarding the incidence and severity of the morbidity of radiation therapy and of progressive prostate cancer, it is necessary to examine the role of RT in locally advanced prostate cancer through a prospective randomized study comparing hormonal therapy versus combined hormonal and radiation therapy.

At the ASTRO conference held in Boston in October of 2008, Dr A. Widmark and colleagues presented the results of a trial that was similar to PR.3 titled SPCG-7 [*Widmark, 2009*]. The SPCG-7 trial randomized patients with intermediate and high-risk prostate cancer to radiation therapy with or without continuous hormonal therapy using long term antiandrogens rather than LHRH agonists. The primary endpoint of the study was cause specific survival and, with a median follow-up of 7.5 years, the investigators reported a 12% improvement in death from prostate cancer and a 9.8% improvement in overall survival at 10 years. In light of the SPCG-7 results described above, disease specific survival as a secondary endpoint is included as a means of further exploring the impact of pelvic radiotherapy on the survival of the PR.3 study population.

A quality of life component will be used to evaluate differences in the two arms of the study which may add additional information that routine treatment-related toxicity may fail to identify. It will be important to evaluate quality of life over the duration of the study as delayed toxicity may be the sole difference in outcome between the two arms. North American centres will be randomized to one of two quality of life instruments -- either QLQ-C30+3 and trial specific checklist (PR17) <u>or</u> FACT-P. All MRC-UK centres will use the FACT-P instrument unless the UK trial coordinator has given a site permission to opt out.

The objective of the quality of life assessment in this study is:

1. to provide a description of quality of life of patients with prostate cancer treated with hormones from the time of diagnosis to death
2. to determine whether addition of prostate irradiation affects patients' quality of life
3. to assess responsiveness to change of two quality of life instruments - QLQ-C30 plus PR.17, and FACT-P.

The EORTC QLQ-C30+3 is a "core" questionnaire consisting of 33 items that assess five functional dimensions (physical, role, cognitive, emotional and social), three symptom dimensions (e.g. fatigue, pain, nausea/vomiting) and global quality of life. The core questionnaire, known as the QLQ-C30, has been psychometrically validated in patients with lung cancer. Three items have been added which are of a developmental nature. The prostate cancer checklist (PR17) consists of additional items that provide additional details on symptom-related distress and was specifically designed for this study. Five additional questions (the "subjective significance" module) were designed to elicit the patient's opinions about the perceived significance of changes in physical function, emotional state, social interactions, overall physical discomfort, and overall quality of life.

FACT-P is a quality of life instrument derived from FACT (Functional Assessment of Cancer Therapy), a 33 item general cancer quality of life measure that was developed and validated in 854 patients with various cancer diagnoses *(Cella, 1993 (a))*. It assesses five domains: physical, social, emotional and functional wellbeing and relationship with doctor. This instrument was adapted for prostate cancer by the addition of 12 items of specific relevance to patients with tumours of the prostate.

Although there is evidence for validity of both of these quality of life instruments, neither one has been proven in a trial to be responsive to change over time in patients with carcinoma of the prostate. In this study, the sample size estimated for the primary outcome is much greater than that necessary to detect quality of life differences between these two study arms. A randomization between two different instruments and comparison of responsiveness to change of two quality of life instruments, QLQ-C30+3 and trial specific checklist (PR17) and FACT-P, will therefore be possible. For reasons of compliance and clarity of instructions, centres will be randomized, rather than individual patients. A centre will be assigned randomly to use either FACT-P or QLQ-C30+3 and trial specific checklist (PR17) for all of their patients on both study arms.

3.0 BACKGROUND THERAPEUTIC INFORMATION

3.1 Anti-Androgens

3.1.1 Flutamide

Flutamide is a potent inhibitor of androgen uptake in endocrine-target tissues. Its major metabolite is the alpha hydroxylated derivative and both compounds have a plasma half-life of 5 to 6 hours. Twenty-eight percent of the drug is excreted in the urine within 24 hours.

By elevating the plasma testosterone and estradiol levels, flutamide can cause fluid retention but the major toxicity, which has been fatal, is hepatic failure. The development of pruritus, dark urine (the urine can be coloured amber or yellow-green anyway from the drug) or jaundice is an indication that the drug should be discontinued and periodic liver function tests as a routine are recommended. The hepatic damage is usually reversible.

Gynecomastia and/or breast tenderness, sometimes with galactorrhea, can develop. These usually disappear upon discontinuation or a reduction in dosage. (These symptoms do not disappear while continuing treatment.)

Flutamide can cause nausea, vomiting, an increase in appetite, diarrhea or rarely, anorexia and constipation.

Also rare are thrombophlebitis, pulmonary embolism and myocardial infarction.

Insomnia and lassitude are more common than headache, blurred vision and decreased libido which occur rarely.

Flutamide is available as 250 mg yellow tablets (Euflex, Canada and Drogenil, UK) and 125 mg capsules (Eulexin, USA) and will be administered as 250 mg po TID. Store at room temperature.

3.1.2 Nilutamide

Nilutamide is a non-steroid that specifically blocks androgen-binding receptors. When used in conjunction with orchiectomy it has improved survival in advanced cases (compared to orchiectomy alone).

Nilutamide can interact with warfarin anticoagulants, phenytoin, propranolol, chlordiazepoxide, lidocaine, diazepam and theophylline, increasing blood levels and therefore requiring dosage adjustment. Patients should be warned against alcohol consumption because of a possible disulfiram-like reaction.

An increase in visual adaptation time when passing from light to darkness is one of the most frequently reported adverse effects. This can decrease, even with continued treatment, and can be ameliorated with the use of sunglasses. Patients must take care when driving or operating machinery. Other visual disturbances seen are impaired vision of colours and, rarely, blurred vision, photophobia and dazzling.

Complaints of hot flushes, decreased libido and impotence are frequent but these reports have been in conjunction with castration.

Nausea, vomiting, constipation, rarely diarrhea and gastric pain have been reported.

Interstitial pneumonitis has been reported in 2% of patients exposed to nilutamide in controlled clinical trials (*Aventis Pharmaceuticals Inc., Oct. 2000*). As well, tachycardia, hypertension and rashes have occurred rarely. Nilutamide is contraindicated in patients with

severe hepatic impairment, in patients with severe respiratory insufficiency and in patients with hypersensitivity to nilutamide or any components of this preparation.

Nilutamide (Anandron), as an optional alternative for flutamide would be administered at 100 mg, three times a day for one month and then 150 mg, once a day. It is commercially available in 50 mg and 100 mg tablets. Store at room temperature.

3.1.3 Bicalutamide (Casodex)

Bicalutamide is a potent nonsteroid antiandrogen without androgenic or progestational properties. The chemical name is Propanamide, N-(4-cyano-3 (trifluoromethyl) phenyl)-3-((4-fluororphenyl) sulphonyl)-2-hydroxy-2-methyl, (+/-). It is a racemic mixture, the antiandrogen activity residing exclusively with the (-) or (R) enantiomer.

Gynecomastia with breast tenderness, hot flashes and pruritus are the most frequent adverse effects of the drug. Other toxicity directly related to the pharmacological effect of the drug is infrequent. Asthenia, pelvic pain, peripheral edema, diarrhea, rash, constipation, impotence, dyspnea, nausea, vomiting and pain have all been recorded. No change in cardiac status during long-term administration of bicalutamide with at least 50 mg daily has been observed.

Since transaminase abnormalities and rarely jaundice have been reported with the use of bicalutamide, periodic liver function tests should be considered. Abnormalities are usually reversible upon discontinuation of drug.

In bicalutamide monotherapy at 150 mg daily only 2.9% of subjects were withdrawn because of treatment-related toxicity (although 5.2% were withdrawn in total). The principal adverse events were gynecomastia (26%), breast pain (32%) and hot flashes (9.2%) although about 2% experienced nausea, diarrhea and/or constipation. Changes in liver function are infrequent (< 2%) and have resolved despite continuing therapy, although in a few cases, jaundice has occurred for which bicalutamide-induced hepatotoxicity cannot be excluded.

Bicalutamide (Casodex) is available as white tablets containing 50 mg of micronized drug. Store at room temperature in a dry place. Bicalutamide (Casodex) as an optional alternative for flutamide would be administered 50 mg PO as a once daily dose.

3.2 LHRH Agonists

Short-term worsening of signs and symptoms associated with prostate cancer such as increased bone pain or urinary obstruction have been reported at the initiation of therapy. This is unlikely after prior anti-androgen treatment. This could contribute in some patients (e.g. those with metastatic vertebral lesions or a history of urinary obstruction) to paralysis or renal failure with or without fatal complications.

Therefore it is recommended that patients are treated with an oral anti-androgen for at least 2 weeks beforehand to counter any "flare" phenomenon. The patient may or may not then continue the anti-androgen at the physician’s discretion.

3.2.1 Goserelin (Zoladex)

Goserelin is a synthetic decapeptide analog of gonadotrophin releasing hormone. Administered chronically, it inhibits gonadotrophin production thus resulting in testicular regression. The

serum testosterone falls to the levels seen in surgically castrated men within three weeks and is maintained at these levels as long as therapy is continued.

Patients frequently (> 50%) experience hot flushes, decreased libido and erections, and less frequently, (< 10%), gynecomastia and local pain at the injection site.

Rarely (< 1%) cardiovascular or cerebrovascular accidents, skin rashes and hematological complications have been noted.

Goserelin depot is released continuously over at least 28 days (Zoladex) or 3 months (Zoladex LA) from a cylindrical rod of biodegradable copolymer. It is supplied as a sterile ready-to-use syringe for subcutaneous use, the needle containing 3.6 mg of goserelin (Zoladex) or 10.8 mg of goserelin (Zoladex LA) in the D-L Lactide-glycolide copolymer rod. Protect from light and moisture. Store in intact package below 25°C.

3.2.2 Leuprolide (Leupron) [Leuprorelin in U.K.]

Leuprolide is a synthetic nonapeptide analog of gonadotrophin releasing hormone. Chronic administration results in inhibition of gonadotrophin production, thus inhibiting steroid hormone production from the testis. There is a transient increase in testicular steroids at the commencement of treatment. Intramuscular injection of the depot preparation prolongs plasma levels of leuprolide for a month.

Decreased testicular size, hot flushes, impotence, gynecomastia and a decrease in libido are all related to hormonal changes consequent to the primary effect of the agent. The reversibility of the clinical symptoms of hypogonadism that occur has not been established.

Other serious adverse effects that have been reported are: congestive cardiac failure, EKG changes, hypertension and hypotension, myocardial ischemia and infarction, thrombosis, pulmonary embolism and strokes. The drug literature lists a large number of other associations.

Leuprolide is given intramuscularly every 28 days (Leupron Depot) or every 3 or 4 months (Leupron Depot LA). The injection site should be varied periodically. Each single dose vial of Leupron Depot contains 7.5 mg (Leuprorelin 3.75 mg UK) or Leupron Depot LA contains 22.5 mg (Leuprorelin 11.25 mg UK) or Leupron Depot 30 mg of leuprolide acetate incorporated as sterile biodegradeable lyophilized microspheres, purified gelatin polyactic/polyglycolic acid and D-mannitol. The accompanying ampoule of diluent contains carboxymethylcellulose sodium 5 mg, D-mannitol 50 mg, 1 mg polysorbate 80 and water for injection.

Reconstitute each vial with 1 ml of the special diluent. Discard the remaining, unused diluent.

3.2.3 Buserelin (Suprefact)

Buserelin Acetate is a synthetic peptide analogue of natural gonadotrophin releasing hormone. Chronic administration results in inhibition of gonadotrophin production, thus inhibiting steroid hormone production from the testis. There is a transient increase in testicular steroids at the commencement of treatment. Intramuscular injection of the depot preparation prolongs plasma levels of leuprolide for a month.

Decreased testicular size, hot flushes, impotence, gynecomastia and a decrease in libido are all related to hormonal changes consequent to the primary effect of the agent. The reversibility of the clinical symptoms of hypogonadism that occur has not been established.

Other serious adverse effects that have been reported are: congestive cardiac failure, EKG changes, hypertension and hypotension, myocardial ischemia and infarction, thrombosis, pulmonary embolism and strokes. The drug literature lists a large number of other associations.

Buserelin (as Suprefact Depot) is given subcutaneously every 8 weeks into the skin of the anterior abdominal wall. It is supplied as a sterile ready to use syringe for subcutaneous use, the needle containing 6.3 mg of Buserelin in the two D, L lactide-co-lactide rods. Likewise, Buserelin may also be given subcutaneously every 12 weeks at a dose of 9.45 mg. Protect from light and moisture. Store in intact package below 25°C.

4.0 TRIAL DESIGN

This is a multi-centre non-blinded randomized trial of NCIC CTG, CUOG, SWOG and MRC UK.

Arm A	Bilateral orchiectomy or LHRH agonist (Goserelin, Leuprolide or Buserelin) (patient's choice). Oral antiandrogen (Flutamide, Nilutamide or Bicalutamide) for minimum 2 weeks of LHRH agonist given, otherwise optional.
Arm B	Arm A plus radiation therapy 65-69 Gy (35 to 37 treatment days)

4.1 Stratification

Patients will be stratified to ensure balance in the two study arms of potential prognostic factors. A minimization procedure for treatment assignment as described in Pocock and Simon *(Pocock & Simon, 1975)* will be used in this study. Patients will be stratified according to:

Centre

Initial PSA level:	< 20 μg/L<br>versus 20 to 50 μg/L<br>versus > 50 μg/L
Choice of hormonal therapy:	Bilateral orchiectomy (+/- antiandrogen) versus LHRH agonist + antiandrogen
Method of lymph node-staging:	Clinical (no CT)<br>versus Radiological (CT negative)<br>versus<br>surgical dissection
Gleason Score:	<8<br>versus<br>8 to 10

Prior hormone therapy (excluding orchiectomy) versus no prior hormone therapy.

The imbalances of the distribution of treatment assignment within the levels of each of the above stratification factors will be minimized by this method.

4.2 Randomization

Patients will have the choice of treatment either by bilateral orchiectomy with or without antiandrogen or by LHRH agonist with at least 2 weeks of antiandrogen.

This choice must be made before randomization.

Patients will be randomized to receive either androgen ablation alone or androgen ablation with radical radiation therapy to a planned sample size of 1200 eligible patients.

Participating North American centres will be randomized to one of the two quality of life instruments -- either QLQ-C30+3 and trial specific checklist (PR17) or FACT-P.

All MRC-UK centres will use the FACT-P QoL instrument unless the MRC-UK trial coordinator has given permission to opt out.

4.3 Inclusion of Women and Minorities

There are no exclusions based on race or ethnicity in this trial. In the NCIC Clinical Trials Group as a whole, 60% of patients have been female and 40% have been male. The female preponderance reflects the number of studies performed in breast cancer. Recruitment to trials for disease sites that involve both males and females has been approximately in proportion to the gender incidence *(Marlin, S)*. Insufficient data has been collected to test a similar relationship for racial/ethnic groups. This study, however, will be presented to patients through the major cancer-treatment institutions of the Canadian provinces, to which all racial/ethnic groups have equal access. The intention, therefore, is to recruit subjects from racial/ethnic groups in close approximation to the incidence of the disease in these groups.

In prostate cancer, previous studies have failed to show any interaction between race and the effect of treatment *(Roach, 1992)*. In particular, there seems to be no significant interaction between the effect of androgen blockade and survival, although black men are known to have a worse overall prognosis *(Crawford, 1990)*. Thus there is no special sample size goal for blacks planned. Retrospective subset analyses by race will be performed in this study, but it is realized that the power of such comparisons will be low.

SWOG participates in the Minority Based CCOP and CTEP Minorities programs.

5.0 STUDY POPULATION

Patients with locally (T3 or T4, N0 or NX, M0 or T2, PSA > 40 μg/L or T2, PSA > 20 μg/L and Gleason ≥8) advanced adenocarcinomas of the prostate.

5.1 Eligibility Criteria

There will be no exceptions to eligibility requirements at the time of randomization. Questions about eligibility criteria should be addressed prior to calling for a randomization.

Patients must fulfill all of the following criteria to be eligible for admission to the study:

5.1.1 A histological diagnosis of adenocarcinoma of the prostate within 6 months of randomization. Pathologic material must have been reviewed by the designated local reference pathologist (LRP) at the treating centre prior to randomization. LRP review is not required for SWOG OR MRC-UK patients.

5.1.2 Clinical stage (T3 and T4, N0 or NX, M0 or T2, PSA > 40 μg/L or T2, PSA > 20 μg/L and Gleason ≥ 8) (see Appendix III for staging). Gleason score must be determined (and confirmed by the local reference pathologist in North America).

5.1.3 If investigated, pelvic lymph nodes must be clinically negative. CT or MRI is mandatory only in patients being treated to the prostate only (see 8.2.3). Any node appearing > 1.5 cm on CT or MRI must have had a negative needle aspirate within 3 months of randomization.

5.1.4 The patient must have a bone scan (with X-rays of any areas of abnormal uptake) reported as being free of evidence of bony metastases within 16 weeks prior to randomization.

5.1.5 The patient must not have had a radical prostatectomy.

5.1.6 If the patient has had a lymph node dissection, it must have been histologically negative and performed within the twelve weeks prior to randomization.

5.1.7 Prior to randomization, the patient must have been evaluated and found eligible for the study by a named radiation oncologist within 4 weeks of randomization (see especially section 5.2.4)

5.1.8 Baseline PSA must be within 12 weeks prior to any hormone therapy, (if a TURP has been performed, the pre-randomization PSA must be obtained more than 21 days after the TURP).

5.1.9 The patient may have received prior hormone therapy during the 12 weeks prior to randomization, provided that:

- a negative bone scan preferably within 16 weeks prior to any hormone therapy but definitely within 2 weeks after starting hormone therapy.
- extrascapsular extension remains palpable on rectal re-exam within 4 weeks prior to randomization

AND

- baseline PSA within 12 weeks prior to any hormone therapy will be reported.

5.1.10 The patient must not have received any cytotoxic anticancer therapy prior to randomization and should not receive any non-protocol anticancer therapy until documented progression of the disease. Patients may however have received treatment with a 5 alpha-reductase inhibitor (e.g. Finasteride) for BPH which must have been discontinued at least 4 to 6 weeks before randomization PSA level.

5.1.11 Patients must have an ECOG performance status of 0, 1 or 2 (Appendix II).

5.1.12 Patients must be less than eighty years old.

5.1.13 The patient has completed the pre-randomization quality of life assessment and is willing and able to complete future assessments in either English or French. Should there be sufficientreason why a patient cannot complete these assessments (illiteracy, loss of sight or other equivalent reasons) the inability to complete these assessments will not make the patient ineligible for the study nor inevaluable for the main objective of the study. MRC-UK centres may opt out of the QOL component of the study with the permission of the MRC-UK trial coordinator.

5.1.14 Haematology and Biochemistry. Laboratory requirements have been done within 4 weeks prior to randomization:

haemoglobin	$\geq$ 100 g/L ($\geq$ 10 g/dL)
WBC	$\geq 2.0 \times 10^9$/L ($\geq 2.0 \times 10^3$/uL)
platelets	$\geq 100 \times 10^9$/L ($\geq 100 \times 10^3$/uL)
SGOT<br>SGPT<br>alkaline phosphatase<br>serum creatinine<br>serum bilirubin	< 2.0 x upper normal limit

5.1.15 As it is unknown/uncertain whether these treatments may affect an unborn child, adequate birth control measures should be used by the participant or his sexual partners while participating on this study.

5.1.16 Patient consent must be obtained according to local Institutional and/or University Human Experimentation Committee requirements. It will be the responsibility of the local participating investigators to obtain the necessary local clearance, and to indicate in writing to the NCIC CTG Clinical Trials Coordinator that such clearance has been obtained, before the trial can commence in that centre. Because of differing requirements, a standard consent form for the trial will not be provided but a sample form is given in section 18.0. The patient must sign the consent form prior to randomization. Please note that the consent form for this study must contain a statement which gives permission for the NCIC CTG and monitoring agencies to review patient records (see section 16.0 for further details).

5.1.17 Patients must be accessible for treatment and follow-up. Investigators must assure themselves that the patients randomized on this trial will be available for complete documentation of the

treatment, toxicity, and follow-up.

5.1.18 Protocol ablative hormone treatment is to begin within 4 weeks of patient randomization (LHRH agonist or bilateral orchiectomy) and, should radiation therapy be allocated, this will be started within 8 weeks of randomization.

5.2 Ineligibility Criteria

5.2.1 A history of previous or concurrent malignancy other than non-melanoma skin cancer within 5 years of diagnosis of the prostatic cancer.

5.2.2 The presence of small-cell or transitional-cell carcinoma in the biopsy specimen.

5.2.3 Any prior treatment for carcinoma of the prostate apart from trans-urethral resection.

5.2.4 Any contraindication to pelvic radiotherapy: e.g. previous pelvic radiotherapy, inflammatory bowel disease or severe bladder irritability.

5.2.5 Any serious non-malignant disease resulting in a life expectancy of less than 5 years.

6.0 PRE-TREATMENT EVALUATION

(See Appendix I)

Investigations		Timing
History and Physical Exam including:	performance status<br>Rectal exam (negative nodes)	within 4 weeks prior to randomization
Hematology	CBC, WBC, platelets	
Biochemistry	SGOT, SGPT, alkaline phosphatase, total bilirubin, serum creatinine	
Other Laboratory Tests	Serum testosterone (optional)	
	PSA**	within 12 weeks prior to randomization
Radiology	Bone scan	within 16 weeks prior to randomization
	Chest x-ray (PA and lateral)<br>CT <u>or</u> MRI of abdomen and pelvis (only required if XRT is to be confined to the prostate - see 8.2.3)	within 3 months prior to randomization
Other Investigations	Biopsy (local pathology review required for North American patients)	within 6 months prior to randomization
Toxicity *<br>(include libido + potency)	baseline toxicity evaluation (to document baseline symptoms)	within 4 weeks prior to randomization
Quality of Life	QLQ-C30+3+PR17 or FACT-P***<br>MRC-UK centres will use FACT-P instrument	

* Toxicities will be recorded and graded according to the NCIC CTG Expanded Common Toxicity Criteria (Appendix V).

** If a TURP is performed, the pre-randomization PSA must be obtained no sooner than 21 days after the TURP.

*** North American centres randomly assigned to administer either QLQ-C30+3+PR17 or FACT-P.

7.0 ENTRY/RANDOMIZATION PROCEDURES

7.1 Entry Procedures

7.1.1 NCIC CTG Institutions:

All eligible patients enrolled on the study by a participating treatment centre will be entered into a patient registration log provided by the NCIC CTG. This will automatically provide a serial number for that patient which should be used on all documentation and correspondence with the NCIC CTG.

All randomizations will be done centrally by the NCIC CTG and will be obtained by calling the NCIC CTG Clinical Trials Assistant at (613) 533-6430 or faxing the eligibility checklist to (613) 533-2941. At the time of randomization, a copy of the completed and signed eligibility checklist must be available.

The following information will be required:

- trial code (NCIC CTG Trial PR.3)
- treatment centre and investigator
- confirmation of current REB/IRB approval
- patient's initials, hospital number and NCIC CTG serial number
- confirmation of the requirements listed in Section 5.0, including dates of essential tests and actual laboratory values
- completed and signed eligibility checklist
- stratification parameters

7.1.2 SWOG Institutions: Registration/Randomization:

Investigators will call the Southwest Oncology Group (SWOG) Data Operations Centre c/o Cancer Research and Biostatistics (CRAB) at 206-652-2267 between the hours of 6:30 a.m. and 4:00 p.m. (PT) Monday through Friday. The SWOG Data Operations Centre c/o CRAB will obtain and confirm all eligibility criteria and information as per Sections 5.1 and 5.2. In addition, The SWOG Data Operations Centre c/o CRAB will request the date the informed consent was obtained and the date of IRB approval for each entry. The SWOG Data Operations Centre c/o CRAB will then contact the NCIC Clinical Trials Group (CTG) to register the patient after which the SWOG Data Operations Centre c/o CRAB will contact the institution to confirm registration. SWOG members should not contact the NCIC CTG directly. The NCIC CTG will forward a confirmation of treatment assignment to the SWOG Data Operations Centre c/o CRAB for routing to the SWOG participating institution. Please note: SWOG institutions will follow their normal procedures for documentation of IRB approval.

7.1.3 MRC-UK

This trial has received MREC approval. However, MRC centres must attain LREC approval before randomising patients into the trial. If you would like an LREC submission pack, please contact the MRC Clinical Trials Unit.

Once patient eligibility has been confirmed and consent has been given, the clinician should contact the MRC Clinical Trials Unit to randomize the patient. The data on the Randomisation Checklist (page 1 of the Case Report Forms) will be required during process. Randomisations may be undertaken via:

Cancer Division
MRC Clinical Trials Unit
5 Shaftesbury Road
Cambridge
CB2 2BW

Tel: (01223) 322 000 [Mon-Fri 09h00 - 17h00 only]
Fax: (01223) 311 844

Trial number and treatment will be given over the telephone and confirmed by post shortly after randomisation. Copies of the completed Randomisation Checklist form the Book of Forms should be returned to the MRC Clinical Trials Unit immediately. A further Book of Forms will be dispatched.

Note: The validity of results depends on the authenticity of an the follow-up of all patients entered into the trial. Under no circumstances, therefore, may an allocated patient be withdrawn prior to final analysis, except on disclosure of initial ineligibility.

All randomised patients are to be followed-up until death.

7.2 Stratification

Patients will be stratified according to:

- centre
- PSA < 20 μg/L, 20-50 μg/L, > 50 μg/L
- hormonal therapy: bilateral orchiectomy or LHRH agonist
- method of lymph node staging (clinical or radiological or surgical)
- Gleason score < 8 or 8-10
- prior hormone therapy (excluding orchiectomy) versus no prior hormone therapy.

7.3 Randomization:

Randomization will be given by telephone and confirmed by mail.

NOTE: The validity of results of the trial depends on the authenticity of and the follow-up of all patients entered into the trial. Under no circumstances, therefore, may an allocated patient be withdrawn prior to final analysis, except on disclosure of initial ineligibility.

All eligible patients admitted to the trial will be followed by the coordinating centre. It is the responsibility of the physician in charge to satisfy himself or herself that the patient is indeed eligible before requesting randomization.

All randomized patients are to be followed until death. The follow-up requirement for ineligible patients is minimal follow-up using a Form 5M.

8.0 TREATMENT PLAN

Arm A	Bilateral orchiectomy or LHRH agonist (Goserelin, Leuprolide/Leuprorelin or Buserelin) (patient's choice). Oral antiandrogen (Flutamide, Nilutamide or Bicalutamide) for minimum 2 weeks of LHRH agonist given, otherwise optional
Arm B	Arm A plus radiation therapy 65-69 Gy (35 to 37 treatment days)

Although the NCIC Clinical Trials Group acts as the coordinating agency for the trial, the responsibility for treatment of patients rests with the individual investigator.

Protocol ablative hormonal treatment is to begin within 4 weeks of patient randomization (LHRH agonist or bilateral orchiectomy). Radiation therapy, if so allocated, must commence within 8 weeks of randomization.

8.1 Hormonal Treatment Plan

All patients in this study will receive total androgen blockade. Patients must choose either bilateral orchiectomy or one of the LHRH agonists prior to randomization.

8.1.1 Antiandrogens (Flutamide, Nilutamide, Bicalutamide see 3.1)

Patients in this study (optional for those choosing bilateral orchiectomy) will receive an antiandrogen for at least 2 weeks to counter any "flare" phenomenon. After this time the patient may or may not continue the antiandrogen at the physican's discretion.

The antiandrogen must commence within two weeks after randomization.

8.1.2 LHRH agonists (Goserelin, Leuprolide/Leuprorelin, Buserelin see 3.2)

Except in patients who choose bilateral orchiectomy, within four weeks after randomization all patients shall commence one of the LHRH agonists listed above at the protocol prescribed dose, route and interval of administration. (see section 3.2)

The LHRH agonist must be given continuously until progression (section 10.2) at the protocol prescribed dose, route and interval.

If the LHRH agonist is discontinued because of intolerance or non-compliance, bilateral orchiectomy is recommended to maintain androgen blockade.

8.1.3 Bilateral Orchiectomy

When chosen for ablation, bilateral orchiectomy should be performed within 4 weeks of randomization.

8.1.4 Dose Modification

Drug Dose escalation is not allowed and dose reduction should not be necessary in this study (but may be carried out to ensure patient tolerance at the investigator's discretion, if duly reported together with the reason).

Alternate antiandrogen (see 3.1) should be substituted or antiandrogen should be discontinued and not be restarted should a patient develop dark urine and/or pruritus and hepatic damage confirmed by jaundice or an increase in any liver function test of greater than 3 times the upper limit of normal.

Antiandrogen therapy should be discontinued in cases of unacceptable toxicity.

8.2 Radiation Therapy (ARM B ONLY)

Radiotherapy must commence within 8 weeks of randomization.

8.2.1 Quality of the Radiation

Megavoltage equipment with an effective photon energy of 6 MV or greater and a minimum source-to-axis distance of 100 cm is required.

8.2.2 Number and Arrangement of Beams

A four-field box technique is recommended. A three-field arrangement, four field oblique, rotational technique or five field technique is acceptable.

Specific instructions must be given on the prescription sheet to treat with as full a bladder as possible.

Patients may be treated in the supine or prone position.

8.2.3 The Target Volume

Simulation is part of the treatment process. At this time, the rectum should be opacified with barium and, unless a CT treatment plan is utilized, a Foley catheter with contrast should be inserted to adequately identify the bladder neck.

Patients who have had a previous pathologically negative lymph node dissection will receive the entire 65 to 69 Gy radiation dose to the specific prostate volume (see below).

All other patients will receive 45 Gy to the whole pelvis to a volume encompassing the prostate, the bladder, the anterior portion of the rectum and the internal and external iliac lymph nodes. If the treating physician determines that whole pelvic RT is inappropriate for the patient (ie: patient is unfit) RT may be given to the prostate only.

This will be followed by a boost of 20 to 24 Gy to a specific prostate volume which will include the prostate and the peri-prostatic tissues with any known areas of tumour extension, such as the seminal vesicles, included with a further 1.5 cm margin. The use of CT plans, retrograde urethrograms or placement of marker seeds at the prostatic apex under ultrasound is recommended during planning.

8.2.4 Radiation Fields

Field borders are to be defined at the 50% isodose and the field size is specified as that at the skin for constant source-skin distance techniques or as that at the axis for source-rotation axis techniques.

Whole pelvis fields

The borders for the anterior/posterior whole-pelvis fields shall be:

Superior border: The middle of the S1/S2 interspace as viewed on the lateral projection (Allowance +/- 2 cm).

Inferior border: The bottom of the ischial tuberosities or 1.5 cm inferior to the apex of the prostate, whichever is the lower (Allowance + 1.5 cm; - 0.5 cm).

Lateral borders: 1.5 cm lateral to the pelvic brim at its widest portion (Allowance + 1 cm; -1 cm)

The borders for the lateral whole-pelvis fields shall be:

Superior border: As for the anterior/posterior fields.

Inferior border: As for the anterior/posterior fields.

Anterior border: Mid-symphysis (Allowance + 1 cm; - 0.5cm)

Posterior border: This border should include (at a minimum) the anterior half of the rectum (as demonstrated by the rectal radiological contrast) along that portion of the rectum that is adjacent to the prostate at the midpoint (superior-inferior) of the fields of the specific prostate volume (Allowance + 1 cm; - 0.5 cm).

Fields for the specific prostate volume

The aim is to include the prostate, the periprostatic tissues and all known areas of tumour extension by a margin of at least 1.5 cm.

The borders for the anterior/posterior prostate fields shall be:

Superior border: A minimum of 9 cm above the bottom of the ischial tuberosities (Allowance + 2 cm; - 0.5 cm).

Inferior border: As for the whole pelvis fields.

Lateral borders: A minimum of 4.5 cm lateral to the pubic symphysis (Allowance + 2 cm; - 0.5 cm).

The borders for the lateral prostate fields shall be:

Superior border: As for the anterior/posterior fields.

Inferior border: As for the anterior/posterior fields.

Anterior border: As for the whole-pelvis fields.

Posterior border: As for the whole-pelvis fields.

8.2.5 Beam Modification Devices

Wedges should be used when necessary to improve the dose distribution.

Secondary shielding to the lateral fields is encouraged and is allowed to the anterior/posterior prostate boost fields in order to minimize the amount of normal bladder and/or rectum included in the treated volume, providing that a 2 cm margin is maintained around the target volume (not the known tumour itself).

8.2.6 Dose Distribution and Prescription

Cross-sectional, co-planar beam dose-distributions are required for each treatment distribution to be used, demonstrating the anatomical arrangement of isodose contours around the target volumes. Homogeneity must be +/- 5% of the chosen target dose within the target volume using ICRU Report 50 conventions.

8.2.7 Radiotherapy Toxicity

The toxicities are:

* diarrhea
* fatigue, anorexia
* nausea (rarely vomiting)
* bladder irritation, urinary frequency, urgency, nocturia, obstruction
* rectal irritation and/or ulcer - pain, bleeding and mucous
* myelosuppression
* alopecia (radiated area), redness, skin sensitivity

Therapy interruptions should be kept to a minimum and not longer than one week when at all possible. The size of the individual fractions (180 or 200 cGy depending on the phase of treatment) must not be varied and the requisite number should be completed in 49 treatment days or as short a time thereafter as possible.

8.2.8 Real-time Review (for North American patients)

For efficiency in review, it is preferred that all plans for the entire treatment be sent together for real-time review. It is understood however, that this may not always be possible and it is then requested that the boost-plan documentation be sent later but as soon as it is available.

The prescription sheet(s) must show that:

* All fields must be treated at every fraction.
* The dose per fraction to the target volume must be 180 cGy for the whole-pelvis volume and 200 cGy for the specific prostate volume.
* Five fractions are to be delivered per treatment week.
* The total dose to the whole-pelvis volume is 45 Gy.
* The additional dose to the specific prostate volume is 20 to 24 Gy.
* If the specific prostate volume alone is to be treated (patients with negative node dissections) the total dose is 65 to 69 Gy in 35 to 37 fractions. This allows individual radiation oncologists to choose fraction sizes between 180 cGy and 200 cGy as long as the criteria of the previous sentence are met.

The following documentation with the content specified is required for real-time review. It should be forwarded by courier to:

Dr. Charles Hayter
c/o Lori Livingstone
Clinical Trials and Epidemiology Department
Toronto Sunnybrook Regional Cancer Centre
2075 Bayview Ave., Toronto, ON M4N 3M5

usually before treatment has commenced and in any case no later than three days after the onset of radiotherapy.

Please submit a Form 4.1 with each submission of material for real-time review.

SWOG Institutions: will follow NCIC CTG guidelines for real-time review. Documentation is to be submitted directly to Dr. Charles Hayter at the address above.

Dose distributions of the target volumes, both showing:

* the dose distribution with the prescription point clearly indicated
* the beam arrangements with clear indications of all beam-modifying devices and all calculations relating to these must be attached.

Treatment Prescription Sheets for the Whole-pelvis and Specific-prostate volumes indicating:

* That all fields will be treated at each fraction
* That the treatment will be made with a full bladder
* That the treatment will be given on five days per week
* The appropriate fraction size (180 cGy or 200 cGy)
* The appropriate total dose for each target volume

Simulator films

A "double exposure" technique showing surrounding anatomical landmarks is preferred. If possible, the film should include a margin of 4 cm around the prescribed field border. If polaroids are employed, it is recommended that the photographs be taken at a distance close enough to fill up the entire polaroid print.

* of every actual treatment field with every border of each field outlined by wires representing the 50% isodose of the field. A scale or grid must be imposed on the film and any shielding clearly indicated.

8.2.9 Final Review (for North American patients)

After the completion of radiotherapy, Form 4.2 and copies of all completed prescription sheets should be sent for review. They will be checked to ascertain that the treatment was delivered according to protocol with respect to: all fields treated at every fraction, adherence to correct fraction size and what degree of compliance was attained with regard to total treatment time.

SWOG Institutions: should follow NCIC CTG guidelines for final review. Documentation is to be submitted directly to the NCIC CTG central office.

8.3 Concomitant Therapy

8.3.1 Permitted

Patients will receive ongoing supportive and palliative care (nutritional support, pain control) as indicated throughout the study. Use of prophylactic antimicrobial therapy will be left to the discretion of the attending physician.

8.3.2 Not Permitted

Use of other anti-cancer therapy is not allowed unless documentation of disease progression (including relapse from remission) has occurred.

9.0 EVALUATION DURING STUDY

All patients entered on study must be evaluated according to the schedule outlined in Appendix I with documentation submitted according to the schedule in Appendix IV.

<table>
<tr><th colspan="2">Investigations</th><th>Timing From Randomization</th><th>At Relapse</th></tr>
<tr><td>History and Physical Exam including:</td><td>Performance status^{+}
Rectal exam</td><td rowspan="4">6 months post randomization; then q 6 months for 2 years and then annual until death</td><td>✓</td></tr>
<tr><td>Hematology</td><td>CBC, WBC, platelets</td><td>✓</td></tr>
<tr><td>Biochemistry</td><td>AST (SGOT), ALT (SGPT)****, alkaline phosphatase, total bilirubin, serum creatinine</td><td>✓</td></tr>
<tr><td rowspan="2">Other Laboratory Tests</td><td>* PSA</td><td>✓</td></tr>
<tr><td>Serum testosterone (optional)</td><td>6 months post randomization; then annually</td><td>✓</td></tr>
<tr><td>Radiology</td><td>Chest x-ray (PA and lateral)
Bone scan
CT <u>or</u> MRI of abdomen and pelvis</td><td colspan="2">if indicated</td></tr>
<tr><td>Other Investigations</td><td>Biopsy</td><td>at 5 yr., if in remission (optional)</td><td>✓**</td></tr>
<tr><td>Toxicity ***
(including libido + potency)</td><td></td><td rowspan="2">on the last day of radiation therapy; 6 months post randomization; q 6 months x 2 years; then annual until death</td><td>✓</td></tr>
<tr><td>Quality of Life</td><td>QLQ-C30+3+PR17 <u>or</u> FACT-P (see App. VII)
(FACT-P for MRC-UK)</td><td>✓</td></tr>
<tr><td colspan="4">* Note: confirmatory repeat for PSA > 10. ng (see section 10.2.1).
** Biopsy at relapse if proceeding to radiation therapy.
*** Toxicities will be recorded and graded according to the NCIC CTG Expanded Common Toxicity Criteria (Appendix V).
**** Liver function tests only required with long term anti androgen therapy.</td></tr>
<tr><td colspan="4"><u>SWOG Forms Submission</u>: Group members, CCOPs and CGOPs must submit <u>two</u> copies of all data forms at the required intervals to:
SWOG Data Operations Center
c/o Cancer Research & Biostatistics
1730 Minor Ave. STE 1900
Seattle, WA 98101-1468

Include the NCIC CTG protocol number and patient serial number as well as the SWOG study number and patient number. The SWOG Data Operations Center c/o Cancer Research & Biostatistics will forward the forms to the NCIC CTG.

<u>MRC-UK Forms Submission</u> Forms should be completed and returned to:
PR07
Cancer Division
MRC Clinical Trials Office
5 Shaftsbury Road
Cambridge
CB2 2BW, U.K.

The required forms are attached to the protocol. Additional forms packets will not be supplied when patients are randomized. It is the responsibility of the participating institution to maintain a supply of available forms for data submission.</td></tr>
</table>

10.0 CRITERIA FOR MEASUREMENT OF STUDY ENDPOINTS

Both survival and time-to-progression are important endpoints in this study. It is vital that they be adequately and precisely documented.

10.1 Overall Survival

The primary endpoint in this study is survival defined as the time from randomization to the time of death from any cause or to the date of last follow-up.

10.2 Disease Specific Survival

This endpoint is defined as the time from randomization to the time a patient dies of prostate cancer or prostate cancer and treatment complications.

10.3 Evidence of Disease Progression

10.3.1 Biochemical Criteria of Progression

Criterion 1: If a serum PSA nadir achieved at any time is ≤4 then biochemical progression would be a subsequent increase of serum PSA to >10.

Criterion 2: If serum PSA never falls to ≤4, then biochemical progression would be a subsequent increase in PSA that is both >10 and at least 20% above the nadir value.

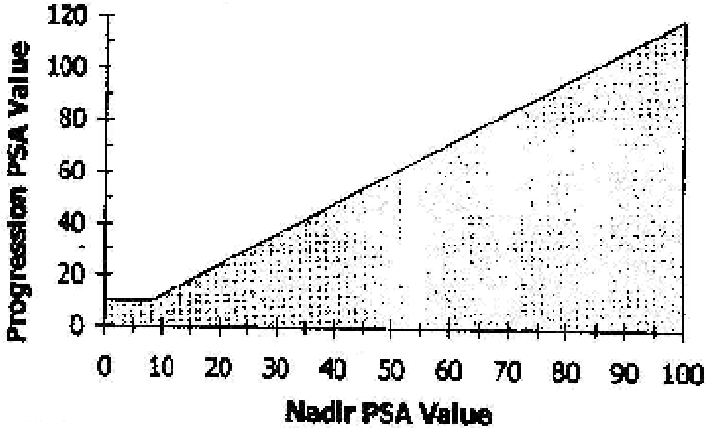

Progression has occurred when one or other of the criteria for serum PSA levels given below has been met on TWO CONSECUTIVE samples. The date of progression is the date of the first such sample meeting the criterion.

Figure 1: Graphical Representation of Criterial 1&2

10.3.2 Radiological and/or Clinical Criteria of Metastases

Radiological evidence of bone and/or parenchymal organ metastases. Radiological and/or clinical evidence of inaccessible lymphadenopathy of > 2 cm in maximum diameter is in itself sufficient to constitute progression.

However, palpable soft tissue masses or superficial lymph nodes should be aspirated for cytological confirmation and, if this is negative, biopsied and histological confirmation of malignancy obtained before the patient is deemed to have progressed.

10.3.3 Local Progression

The development of an obstructed ureter constitutes evidence of progression.

Urethral obstruction or bleeding necessitating a trans-urethral resection constitutes evidence of progression only if resected tissue demonstrates viable malignancy.

Palpable enlargement of an existing abnormality or regrowth of a previously regressed prostate gland constitutes evidence of progression only if accompanied by a biopsy demonstrating viable malignancy.

10.4 Dating of Progression

Biochemical progressions are dated by the day of the first elevated PSA sample (see 10.2.1).

Radiological and/or clinical metastatic progressions are dated by the day of the first abnormal examination, whether or not it is authenticated by histology.

Local progressions are dated by the day of the first abnormal clinical or radiological examination, whether or not any required histological authentication was acquired at that time or only at subsequent surgery.

10.5 Other Endpoints

Symptomatic local control measured as the rate of surgical interventions necessary for symptomatic local disease; the combined incidence of trans-urethral resections, stent insertions, nephrostomies and colostomies.

All patients who have completed the quality of life questionnaires are evaluable for quality of life as measured by the QLQ-C30+3 and trial specific checklist (PR17) or FACT-P.

10.6 Management Following Progression

Following progression, as defined in sections 10.2.1 and 10.2.2, continued androgen suppression, either in the form of bilateral orchiectomy or continuing administration of LHRH agonist, is mandatory. Any additional therapy is at the discretion of the physician.

In the event of local progression (see 10.2.3) in a patient who did not receive pelvic radiation therapy, radiotherapy may now be given with palliative or radical intent, but again, after progression, all treatment is at the discretion of the physician. Should radiotherapy be given at this time, technique and dose are not specified by this protocol.

11.0 ADVERSE EVENT REPORTING

This protocol does not contain investigational agent(s) and toxicities occurring as a result of these commercially available treatments, should be reported in the manner described below. In addition, your local Research Ethics Board (REB) should be notified.

Adverse drug reaction (ADR) reporting should be based on the NCIC CTG Common Toxicity Criteria (see Appendix V).

11.1 Any fatal (grade 5) or life threatening (grade 4) adverse reaction which is due to or suspected to be the result of a protocol drug must be reported to the cooperative group headquarters and to the Study Chair by telephone within 24 hours of discovery. Known/expected grade 4 hematologic toxicities need not be reported by telephone.

11.2 Unknown/unexpected adverse reactions (≥ grade 2) resulting from commercial drugs prescribed in this protocol are to be reported to the co-operative group headquarters, to the Study Chair, and to the IDB (Investigative Drug Branch) within 5 to 10 working days of discovery. Second malignancies unrelated to protocol therapy are no longer reportable as serious adverse events. Unrelated second malignancies will still be collected using the trial specific case report forms.

11.3 Written details will be reported on the standard Adverse Event Report (AER/ADR) form of the group (NCIC CTG AER form for Canada) and the form must be signed by the treating investigator. All ADR reports are to be accompanied by evidence of notification to the institutional REB/IRB.

The written AER/ADR form must be sent to the respective group's office and the NCI Investigational Drug Branch.

NCIC Clinical Trials Group
10 Stuart Street
Queen's University
Kingston, Ontario K7L 3N6
Phone: 613-533-6430
Fax: 613-533-2941

NCI US
Investigational Drug Branch
P.O. Box 30112
Bethesda, MD 20824
Phone: 301-230-2330
Fax: 301-230-0159

The participating groups will call the lead group (NCIC CTG) to report the telephone ADRs.

11.3.1 All Southwest Oncology Group (SWOG) investigators are responsible for reporting of adverse drug reactions according to the NCI and Southwest Oncology Group Guidelines. SWOG investigators must:

Call the SWOG Operations Office at 210/677-8808 within 24 hours of any suspected adverse event deemed either drug-related or possibly drug-related.

Instructions will be given as to the necessary steps to take depending on whether the reaction was previously reported, the grade (severity) of the reaction, study phase, and whether the reaction was caused by investigational and/or commercial agent(s). The SWOG Operation Office will immediately notify the NCIC Clinical Trials Group (CTG).

Within 10 days the investigator must send the completed (original) FDA 3500 Form (for regimens using only commercial agents) to the NCI:

Investigational Drug Branch
P.O. Box 30012
Bethesda, Maryland 20824

In addition, within 10 days the investigator must send:

- a copy of the above report,
- all data records for the period covering prestudy through the adverse event, and
- documentation of IRB notification, to the following address:

ADR Program
SWOG Operations Office
14980 Omicron Drive
San Antonio, TX 78245-3217

At the SWOG Operations Office a multilayered review will be performed and any pertinent finding will be forwarded to the NCIC Clinical Trials Group, NCI, study coordinator, and SWOG Data Operations Center c/o Cancer Research & Biostatistics along with any supporting documentation.

11.3.2 MRC-UK

This protocol does not contain investigational agent(s), and toxicities, occurring as a result of these commercially available treatment, should be reported in the manner described below.

Adverse drug reaction (ADR) reporting should be based on the NCIC CTG Common Toxicity Criteria (appendix V).

Any fatal (Grade 5) or life threatening (Grade 4) adverse reaction which is due to or suspected to be the result of a protocol drug must be reported to the Cancer Division of the MRC Clinical Trials Unit by telephone within 24 hours of discovery. Known/expected Grade 4 haematological toxicities need not be reported by telephone.

11.4 Non-treatment related toxicities. If a toxicity is felt to be outside the definitions listed above and unrelated to the protocol treatment, this must be clearly documented on the case report forms that are submitted to the Data Management Office according to the Data Submission Schedule. This does not in any way obviate the need for reporting the toxicities described above.

11.5 These protocol adverse event reporting directions do not replace, but are in addition to, routine institutional policy for the local reporting of adverse reactions caused by these commercially marketed drugs.

12.0 PROTOCOL TREATMENT DISCONTINUATION

12.1 Criteria for Discontinuing Protocol Treatment

Patients may stop protocol treatment in the following instances:

12.1.1 Intercurrent illness which would, in the judgment of the investigator, affect assessments of clinical status to a significant degree, and require discontinuation of protocol therapy.

12.1.2 Unacceptable toxicity as defined in Section 8.1.4 and 8.2.7 or as determined by the attending physician.

12.1.3 Discontinuation of LHRH agonist therapy is “off protocol treatment” unless bilateral orchiectomy is substituted.

12.1.4 Disease progression as defined in Section 10.0. This section also outlines treatment options at progression.

12.1.5 Request by the patient.

12.1.6 For details of follow-up after progression or stopping protocol treatment, see Section 9.0.

13.0 CENTRAL REVIEW PROCEDURES

13.1 Local Pathology Review

Canadian centres should designate a pathologist to be the PR3 local reference pathologist. S/he must review the biopsy material and allocate a Gleason score prior to randomization. (This review is not required for SWOG or MRC-UK patients.)

13.2 Central Radiotherapy Review

The instructions for real-time review and a list of required material is given in section 8.2.8.

Instructions for the final review are given in section 8.2.9.

14.0 STATISTICAL CONSIDERATIONS

14.1 Objectives and Study Design

The primary objective of this study is to determine whether the addition of external beam radiation to total androgen ablation improves survival in patients with locally advanced prostate cancer. Allocation of the patients to one of the two treatment arms will be performed in such a way that imbalances in the stratification variables will be minimized. The stratification variables include centre, initial PSA level (< 20 μg/L vs 20 to 50 μg/L vs > 50μg/L), hormonal therapy choice (LHRH vs bilateral orchiectomy), method of lymph node staging (clinical vs surgical), Gleason score (< 8 vs 8-10) and prior hormone therapy. A minimization procedure for treatment assignment as described in Pocock and Simon *(Pocock & Simon, 1975)* will be used in this study. The imbalances of the distribution of treatment assignment within the levels of each of the above stratification factors will be minimized by this method.

14.2 Study Endpoints and Analysis

Overall survival, the primary endpoint of the study, is defined as the time from randomization to the time of death from any cause or to the last follow-up. Log-rank statistics will be used to compare the survival experience between the two arms. A Cox proportional-hazards model will be used to assess prognostic factors, and the treatment effect will then be tested after controlling for the important prognostic variables.

Secondary endpoints include disease specific survival, time to progression, the rate of surgical intervention for symptomatic local disease and quality of life scores.

Disease specific survival is defined as the time from randomization to the time a patient dies of prostate cancer or prostate cancer and treatment complications. A log-rank test will be used to test the cause specific hazard, and the Gray test *[Gray, 1988]* will be used to test the cumulative incidences of the cause specific deaths during the trial.

Time to progression is defined as the time from the date of randomization to the date of first documented disease progression (see section 10.3), or the date the patient died of disease or disease and its treatment complication. The analysis that will be performed for this secondary endpoint will be similar to the analysis for overall survival.

The rate of surgical interventions necessary for symptomatic local disease will be summarized by treatment arm.

Since quality of life will be assessed longitudinally, the method of analysis of variance for repeated measures *[Zee, 1991]* will be used to analyze domains represented by aggregate scores. The domains represented by single items are in the form of repeat categorical data, they will be analyzed using the generalized least squares method proposed by Koch et al *[Koch, 1977]*. The profiles of the quality of life scores will be displayed and compared between the two treatment arms.

14.3 Sample Size and Duration of Study

With the advent of widespread PSA screening in the mid 1990’s, patients are being diagnosed earlier with prostate cancer. The 10 year survival for patients with T3 N0 M0 carcinoma of prostate treated with hormonal therapy is estimated to be 48% to 62%. Assuming a 57% survival at 10 years is obtained in the control arm, we will have 80% chance, using an overall 2.5% level one-sided test (0.1% will be spent on the hormone refractory prostate cancer endpoint, see interim analysis for details) of detecting a 8.4% increase in 10-year survival (i.e., a hazard ratio of 1.32) with a total of 1200 eligible patients in the study. A one-sided test of significance is justified by asymmetric nature of the question. Radiotherapy would be added to total androgen ablation in clinical practice if, and only if, there were an increased survival of 8.4% or more in the radiotherapy arm of this study. If the increased survival were less than 8.4% or if the survival in the radiotherapy were equivalent or worse than that in the androgen-ablation-only arm, there is no interest in how much worse the radiotherapy arm is. Assuming an accrual rate of 160 patients per year, we need another 2.5 years to complete accrual to the study. The required number of events (421 deaths) will be met with another 5.8 years of follow-up. Therefore, the total duration of the study is about 16 years. The study event rate will be monitored closely for the duration of the study and analysis of the primary endpoint will be performed once the required number of events is achieved.

14.4 Sample Size for Quality of Life

Two quality of life questionnaires will be used in this study, they are the EORTC QLQ-C30+3 and the FACT-G plus the "additional concerns" subscale for patients with prostate cancer (FACT-P). Centres will be stratified by the expected accrual size before randomization to either EORTC QLQ-C30+3 or FACT. For Canadian centres, the expected accrual size is based on accrual records prior to the start of the study. The US institutions will be classified by their expected accrual size prior to the start of the study. Centres will be randomized to one of the two instruments within each stratum.

In a recently completed NCIC CTG symptom control study comparing antiemetic maintenance therapy using oral ondansetron versus placebo, the EORTC QLQ-C30 nausea and vomiting subscore was significantly different between the two arms (p=0.0008, n=177) with an observed difference of 11% (standard deviation = 25.46). The global quality of life was not significantly different with an average subscore of 52% (standard deviation = 25.37). If global quality of life subscore is considered a major endpoint in this part of the study, we would need an adequate number of subjects to detect at least a 10% difference between the two arms. In order to have 80% power, using a two-sided 5% level test, of detecting a 10% difference in global quality of life, a total of 202 patients are required for the EORTC QLQ measure.

The FACT questionnaire has been fully validated. The FACT-G total score was shown to be able to distinguish metastatic (mean=79.6) from non-metastatic disease (mean=83.7). Both Physical well-being and Functional well-being subscales were shown to have high internal consistency and sensitivity to disease status. The test-retest reliability for all subscales as well as the total score is relatively high (> 0.8). In a validation analysis reported by Cella *(Cella, 1993 (b))*, the Physical and Functional well-being subscales have means of 20.49 (range 0-28) and 17.96 (range 0-28) respectively with standard deviations of 5.45 and 6.10 respectively. The FACT-G total score in the validation study has a mean of 82.06 (range 0-112) with a standard deviation of 15.86. In order to have a 80% power, using a two-sided 5% level test, of detecting a 10% difference for the Functional Well-being subscores, we need a total of 150 patients. The other subscales including the FACT-G total score have a lower coefficient of variation and therefore they require a smaller sample size than the Functional Well-being subscale.

Thus, the EORTC QLQ portion of the quality of life study only requires one sixth of the total sample and the FACT portion requires only 150 patients. Since the projected sample size is 1200 patients for the whole study, we will have more than enough samples for the quality of life questions and will give us more than 80% power to detect the 10% difference with both EORTC QLQ and FACT subscores.

14.5 Safety Monitoring

Toxicities will be monitored on an ongoing basis by the central office and their frequencies reported annually at the investigators' meetings.

14.6 Interim Analysis

It is the responsibility of the NCIC CTG Data Safety Monitoring Committee to carry out the interim analyses and the final analysis from a pooled dataset.

After approximately 750 patients were randomized to the study, the trial committee expressed the desire to include an additional efficacy analysis using the development of hormone refractory prostate cancer (HRPC) as the outcome of interest. This was based on an unconfirmed observation of an imbalance between the study arms regarding the incidence of HRPC. The definition of HRPC that will be used in the analysis is as follows:

1. Biochemical relapse: Two consecutive rises over the previous reference or nadir value. The PSA rise must be at least 2 ng/ml greater than the reference or nadir value. The date of relapse will be the first date this occurs OR
2. Date of distant metastatic disease in the absence of biochemical relapse as defined above OR
3. Date of death due to prostate cancer in the absence of prior biochemical relapse or distant metastatic disease as defined above.

This analysis will be performed with the current dataset. The results will be presented to an independent Data Safety Monitoring Committee (DSMC) for review and consideration. Study closure will be made if a statistically significant difference is seen between the treatment arms with respect to time to development of HRPC (one-sided p < 0.00l log rank test).

In addition to the analysis described above, two interim analyses are planned to allow early termination of the study should an extreme difference in overall survival between the groups develops. The first interim analysis will be performed after we have obtained a total of 141 events (deaths) in the study (i.e., 33% of information). The second interim analysis will be performed after we have obtained 281 events (deaths) in the study. The interim analysis results will be presented to the monitoring committee and early termination will be considered on the basis of the following stopping rule: At the first interim analysis, we would consider stopping the trial if a one-sided p-value of 0.001 is obtained from a log-rank test on the primary endpoint of overall survival in favour of the arm including radiotherapy. For the second interim analysis, we would consider stopping the trial with a one-sided p-value of 0.005. This significance level is based on the type I error spending function as proposed by Lan and DeMets *(Lan & DeMets, 1983)* based on O'Brien-Fleming's design truncated at a significance level of 0.001 (O'Brien, P. C. & Fleming, T. R., 1979). A significance level of 0.022 will be used in the final analysis to maintain the overall study-wise error rate of 0.025 using a Bonferroni adjustment *(Miller, 1981)*.

With the understanding that the MRC will be working from a protocol that differs somewhat in format and procedures, the data management differences (case report form design, data fields unit and interpretation, data collection schedule, serious adverse event reporting, and validation procedure) would not affect the interpretation of the primary questions.

15.0 PUBLICATION POLICY

The final publication policy will be agreed by all participating groups. A draft publication policy is summarized below:

15.1 Authorship of Papers, Meeting Abstracts, Etc.

15.1.1 Prior to trial activation, it will be decided whether to publish the trial under a group title, or with naming of individual authors. If the latter approach is taken, the following rules will apply:

- The first author will generally be the chair of the study.
- A limited number of the members of the NCIC Clinical Trials Group, Southwest Oncology Group and MRC-UK may be credited as authors depending upon their level of involvement in the study.
- Additional authors, up to a maximum of 15, will be those who have made the most significant contribution to the overall success of the study. This contribution will be assessed, in part but not entirely, in terms of patients enrolled and will be reviewed at the end of the trial by the study chair.

15.1.2 In an appropriate footnote or at the end of the article the following statement will be made:

> "A study coordinated by the NCIC Clinical Trials Group. Participating investigators included: (a list of the individuals who have contributed patients and their institutions)."

15.2 Responsibility for Publication

It will be the responsibility of the study chair to write up the results of the study within a reasonable time of its completion. If after a period of six months following the analysis of study results the draft is not substantially complete, the central office reserves the right to make other arrangements to ensure timely publication.

15.3 Submission of Material for Presentation or Publication

Material may not be submitted for presentation or publication without prior review by the NCIC Clinical Trials Group and approval of the trial chair. Individual participating centres may not present outcome results from their own centres separately. Supporting groups and agencies will be acknowledged.

16.0 ETHICAL, REGULATORY AND ADMINISTRATIVE ISSUES

16.1 REB (Research Ethics Board) Approval for Protocols

16.1.1 REB Composition: Research Ethics Boards will be constituted according to local interpretation of the Canadian Medical Research Council (MRC) Guidelines.

Because this trial involves the Southwest Oncology Group, and the NCI U.S., American research ethics boards (REBs) must abide by U.S. Food and Drug Administration (FDA) regulations concerning board membership, as follows: each REB must have at least five members with varying backgrounds, at least one of them nonscientific, and at least one member not otherwise affiliated with the institution; no REB may consist entirely of men or of women, or of members of one profession. (See Protection of Human Subjects, 45 CFR 46 for full statement of requirements.)

Initial Approval
Member centres wishing to participate in a trial are required to obtain local ethics approval of the protocol by the appropriate research ethics board (REB) and to forward this written approval and consent to NCIC CTG..

Continuing Approval
Annual re-approval is required for as long as the trial is open to patient accrual.

Amendments and Revisions
Protocol amendments and revisions will be circulated in standard format with clear instructions regarding REB review if required.

16.1.2 MRC-UK

The trial will be submitted by the MRC to a Multicentre Research Ethical Committee before clinicians can enter patients. Before entering patients into the study, clinicians must also ensure that the protocol has received clearance from their local Ethics Committees. The patient’s consent to participate in the study should be obtained after a full explanation has been given of the treatment options, including the conventional and generally accepted methods of treatment, and the manner of treatment allocation.

The right of the patient to refuse to participate without giving reasons and without prejudicing his further treatment must be respected. After the patient has entered the trial, the clinician must remain free to give alternative treatment to that specified in the protocol at any stage if he/she feels it to be in the patient’s best interest, but the reasons for doing so should be recorded and the patient will need to remain within the study for purposes of follow-up and data analysis according to the treatment.

A statement of MRC policy on the ethical considerations in the clinical study of cancer therapy including questions of informed consent, is available from the CTU Cancer Division and may be used to give guidance to participating investigators and to accompany applications to the local Ethics Committee.

16.2 Informed Consent

Process:

Before recruitment and enrollment into the study, each prospective candidate will be given a full explanation of the study. The informed consent form will be submitted for approval to the Research Ethics Board, Institutional Review Board or Ethics Committee that is responsible for review and approval of the study. Each consent form must include all of the relevant elements required by Canadian authorities.

Once this essential information has been provided to the patient and the investigator is assured that they understand the implications of participating in this study, the patient will be asked to give consent to participate in the study by signing an informed consent. Patients who cannot give informed consent (i.e. mentally incompetent, or physically incapacitated such as comatose patients), are not to be recruited into the study. Patients competent but physically unable to sign the consent form may have this signed by their nearest relative or legal guardian.

Consent Form Content:

The following elements must appear in the consent form: a description of the purpose of the study (indicating, if appropriate, that the drug is investigational); potential side effects; potential benefits; study design; voluntary participation; and confidentiality.

This consent form may be modified to satisfy local REB requirements. However, please note that the consent form for this and all other NCIC CTG studies must contain a statement which gives permission for the NCIC CTG and other sponsoring and monitoring agencies to review patient records.

Sample Consent:

A sample consent is provided in section 18.0 which may be modified to local needs. Please note that all required elements as described above must remain. A French translation is available on request.

16.3 Centre Performance Monitoring

Ineligibility and timeliness are monitored for all centres and the results are reported in the Centre Performance Index. This index is generated twice a year and there are minimum standards for performance.

Centres are required to submit Eligibility Checklist/Form 1 Initial Evaluation Forms and Form 5 Follow-up Reports within the time guidelines specified in Appendix IV (Documentation for Study).

16.4 On-Site Monitoring

In addition to the routine review of case report forms and supporting documents sent to the central office, NCIC CTG site monitoring may be conducted at participating centres in the course of the study as part of the overall quality assurance programme. The monitors will require access to patient medical records to verify the data.

16.5 Case Report Forms

A list of forms to be submitted as well as expectation dates are to be found in Appendix IV.

17.0 REFERENCES

Adolfsson J. Deferred treatment of low grade stage T3 prostate cancer without distant metastases. Journal of Urology 1993; 149:326-329.

American Cancer Society, Cancer Facts and Figures - 1993. CA: A Cancer Journal for Clinicians 1993; 43(1):7-26.

Babaian RJ, Zagars GK, and Ayala AG. Radiation therapy of stage C prostate cancer: significance of Gleason grade to survival. Seminars In Urology 1990; 8(4):225-231.

Bagshaw MA, Kaplan ID, and Cox RC. Radiation therapy for localised disease. Cancer 1993; 71(3):939-952.

Cella DF, (1993) *(a)* Manual: Functional Assessment of Cancer Therapy (FACT) Scales and the Functional Assessment of HIV Infection (FAHI) Scale, Rush-Presbyterian-St. Luke's Medical Center, Chicago, Illinois.

Cella DF, et al. *(b)* The functional assessment of cancer therapy scale: development and validation of the general measure. J Clin Oncol 1993; 11(3):570-579.

Crawford ED, Blumenstein BA, Goodman PG, Davis MA, Eisenberger MA, McLeod DG, Spaulding JY, Benson R, Dorr FA. Leuprolide with and without flutamide in advanced prostate cancer. Cancer 1990; 66(suppl): 1039-1044.

Crawford E, et al. A controlled trial of leuprolide with and without flutamide in prostatic carcinoma. New England Journal of Medicine 1989; 321:419.

Denis L. *(a)* Prostate cancer, primary hormonal management. Cancer 1993; 71(3):1050-1058.

Denis L, et al. *(b)* Goserelin acetate and flutamide versus bilateral orchiectomy: a phase III EORTC trial (30853). Urology 1993; 42(2):119-129.

Duncan W, et al. Carcinoma of the prostate: results of radical radiotherapy (1970-1985). International Journal of Radiation Oncology, Biology, Physics 1993; 26(2):203-210.

Dupont A, Cusan J, et al. Combination therapy with Flutamid and the LHRH Agonist (D - Trp6, des-Gly - NH2) LHRH ethylamide in Stage C prostatic carcinoma. British Journal of Urology 1993; 72:629-634.

Eisenberger M, Crawford ED, McLeod D, Loehrer P, Wilding G, and Blumenstein B. A comparison of bilateral orchiectomy with or without flutamide in stage D2 prostate cancer. Proc Amer Soc Clin Onc 1997; 16: 2a abstract 3

Fellows GJ, et al. Treatment of advanced localised prostatic cancer by orchiectomy, radiotherapy, or combined treatment. A Medical Research Council Study. Urological Cancer Working Party -- Subgroup on Prostatic Cancer. British Journal of Urology 1992; 70(3):304-309.

Gray RJ. A Class of K-Sample Tests for Comparing the Cumulative Incidence of a Competing Risk. The Annals of Statistics 1988; 16(3), 1141-1154

Holtzman M, Carlton CE, and Scardino PT. The frequency and morbidity of local tumor recurrence after definitive radiotherapy for stage C prostate cancer. Journal of Urology 1991; 146:1578-1582.

Koch GG, Landis JR, Freeman JL, Freeman DH, Lehnen RG. A general methodology for the analysis of repeated measurement of categorical data. Biometrics 1977; 33:133-158.

Kostashuk E., 1993, personal communication.

Lan G. and DeMets D. Discrete sequential boundaries for clinical trials. Biometrika 1983; 70, 659-663.

Marlin S. Master's thesis for Queen's University.

Mettlin C, Jones GW, and Murphy GP. Trends in prostate cancer care in the United States, 1974-1990. Observation from the patient care evaluation studies of the American College of Surgeons Commission on Cancer. CA: A Cancer Journal for Clinicians, 1993; 43:83-91.

Miller RG. Simultaneous statistical inference. New York: Springer-Verlag, 1981.

O'Brien PC and Fleming TR. A multiple testing procedure for clinical trials. Biometrics 1979; 35: 549-56.

Pilepich MV et al. Phase III RTOG trial 86-10 of Androgen Deprivation before and during radiotherapy in locally advanced carcinoma of the Prostate. Proc ASCO (17) 308a, 1998.

Pilepich MV, et al. A phase III trial of androgen suppression before and during radiation therapy (RT) for locally advanced prostatic carcinoma, preliminary report of RTOG protocol 8610. Proc Am Soc of Clin Oncol 1993; 12:229.

Pocock and Simon. Sequential treatment assignment with balancing for prognostic factors in the controlled clinical trial. Biometrics 1975; 31:103-115.

Prescribing information, Nilutamide ® (nilutamide) Tablets, Aventis Pharmaceuticals Inc., Bridgewater, New Jersey, NJ 08807, US, October 2000.

Prostate Cancer Trialists' Collaborative Group. Maximum androgen blockade in prostate cancer: an overview of 22 randomized trials with 3283 deaths in 5710 patients. Lancet 1995. 346, 265-269.

Raghavan D. Discussion of Abstract 3 in Plenary Session ASCO May 19th 1997.

Roach III M, Krall J, Keller JW, et al. The prognostic significance of race and survival from prostate cancer based on patients irradiated on Radiation Therapy Oncology Group protocols 1976 to 1985. Int J Radiat Oncol Biol Phys 1992: 24: 441-449.

Robinson MG, EORTC Protocol 30805. A phase III trial comparing orchiectomy versus orchiectomy and cyproterone acetate and low dose stilboestrol in the management of metastatic carcinoma of the prostate. In: Management of advanced Carcinoma of the Prostate and Bladder, Genitourinary Group Mongr 4. Ed: Smith and Pavone-Mancuso; Alan R. Liss: New York. 1988: 101-110.

Smith PH. The case for no initial treatment of localized prostate cancer. Urologic Clinics of North America, 1990; 17(4):827-834.

Statistics Canada. Cancer in Canada (Annual) Canadian Centre for Health Information, 1993.

Widmark A, Klepp O, Solberg A, Damber JE, Angelsen A, Fransson P, Lund JA, Tasdemir I, Hoyer M, Wiklund F, Fossa SD. Endocrine treatment, with or without radiotherapy, in locally advanced prostate cancer (SPCG-7/SFUO-3): an open randomised phase III trial. Lancet 2009. 373(9660):301-8.

Zee B. and Pater J. Statistical analysis of trials assessing quality of life. In: The Effect of Cancer on Quality of Life, Ed. Osoba D, 1991; Ch. 9, CRC Press.

18.0 SAMPLE CONSENT FORM

INTERGROUP (NCIC CTG, CUOG, SWOG and MRC-UK) PHASE III RANDOMIZED TRIAL COMPARING TOTAL ANDROGEN BLOCKADE VERSUS TOTAL ANDROGEN BLOCKADE PLUS PELVIC IRRADIATION IN CLINICAL ADENOCARCINOMA OF THE PROSTATE
NCIC CTG: PR.3, SWOG: JPR.3, MRC-UK: PR07

Purpose and Description of Study

I have been told by my physician that I have prostate cancer. It is unclear how my cancer can be most effectively treated. I understand that there is no standard treatment for prostate cancer at this stage but radiation therapy alone, hormonal therapy, or a combination of these have been used with similar results.

In order to determine the best therapy for this disease the NCIC Clinical Trials Group has organized a study and I have been invited to participate. I have been told that this study will compare two different forms of treatment for my disease and that if I participate, my treatment will be selected randomly and I shall receive either hormone therapy alone or combined hormone therapy and radiation therapy. The chances of my receiving either of the treatments described is equal and I will be told which treatment I have been assigned. If I am assigned to receive hormone therapy alone, I shall be followed closely and if the tumour starts to grow in the pelvis but has not spread elsewhere, I may also then receive radiation therapy.

I understand that the purpose of this study is to determine which of the treatments is most effective in prolonging life and giving patients with my stage of prostate cancer the best possible quality of life.

About 1200 people from the United States, Canada and other countries will take part in this study.

Study Treatment

All patients will receive hormone therapy. This will be a combination of antiandrogen (Flutamide or Nilutamide or Bicalutamide) (pills) PLUS an antihormone called a LHRH agonist (injections of Goserelin or Leuprolide or Buserelin) OR bilateral orchiectomy (surgery). I understand that I will choose between the two types of hormonal treatment - injections or orchiectomy. Orchiectomy is a surgical procedure to remove the testicles and this is performed under general anesthetic. I understand that if I decide to have the LHRH agonist injection instead of the removal of the testicles I will have to go and have an injection given to me (once a month or once every two months or once every three months) for the rest of my life or until this treatment fails. I will take the Antiandrogen pills every day for as long as my physician directs.

I understand that if I am assigned to receive radiation therapy, the radiation treatments will be given five times each week (for 35 treatments) for 7 weeks. The radiation will treat the prostate gland and the lymph nodes around it unless the lymph nodes have been examined at surgery and found to be free of tumour; in which case, only the prostate will be irradiated.

Potential Side Effects and Risks

All treatments have side effects and my doctors will be monitoring me closely to manage any problems that might develop.

Risks and side effects shared by two or more of the LHRH agents in this trial include:

Very Likely (21% or more):
I have been told that side effects that can very likely occur as a result of the hormonal treatment include the following:

- Impotence (unable to have an erection) and reduced sex drive
- Hot flashes
- Nausea, vomiting
- Pain

Less Likely (5-20%):
I have been told that side effects that can less likely occur as a result of hormonal treatment include the following:

- Excessive development of breasts in men, which may include breast tenderness
- Insomnia (difficulty sleeping)
- Lethargy (feelings of tiredness)
- Increase urge to urinate and need to urinate more often
- Altered night vision
- Sensitivity to alcohol
- Decrease size of testicles
- Changes in blood pressure (hypo or hypertension), which has rarely been associated with strokes (Buserelin)
- Dizziness which may include room spinning (vertigo)
- Numbness and tingling in hands and feet
- Local pain, redness and/or irritation at injection sites if I am receiving injections of LHRH agonist
- Constipation
- Scarring in the lungs may occur in patients treated with the drug Nilutamide which causes shortness of breath and coughing. Most cases recover when the drug is stopped but very rarely this may result in death. If I experience any new or worsening shortness of breath, I will report this immediately to my doctor.
- Lack or loss of strength
- Headache and blurred vision
- Muscle pain
- Joint pain
- Bone pain
- Changes in serum lipids and proteins
- Irregular heartbeat
- Appetite changes

Rarely (1-4%):
I have been told that other side effects that can rarely occur as a result of hormonal treatment include the following:

- Retaining an increased amount of water in my body tissues.
- Itchy rash
- Diarrhea
- Weight loss or weight gain
- Liver sensitivity (jaundice) – If it occurs I understand that I should discontinue the antiandrogen pills and contact my doctor immediately
- Elevated liver function tests
- Cough
- Disease flare reaction (increased bone pain, urinary tract obstruction, spinal cord compression) which can cause pain, tingling in the arms and legs, and problems with movement and sensation, rarely including paralysis
- Heart disease including failure and heart attack

Additional risks and side effects associated with LEUPROLIDE that are considered to be *medically important* are:

Less likely (5 – 20%):
- excess sugar in the blood (hyperglycemia)
- neuromuscular disorders

Rarely (1 – 4%):
- visual changes
- gastrointestinal tract bleeding (e.g. in the stomach or intestines)
- loss of memory
- hearing loss or ringing in the ears
- allergic reaction, in severe cases can include anaphylactic shock (anaphylaxis)
- fever

Lung disorders (including inflammation, pneumonia and fluid collection) have been rarely associated with LEUPROLIDE

Additional risks and side effects associated with GOSERELIN that are considered to be *medically important* are:

Rarely (1 – 4%):
- decreased bone marrow function, may reduce number of infection fighting cells
- flu-like syndrome
- infection
- visual changes
- fever
- benign tumour of the pituitary gland (pituitary adenoma)

Additional risks and side effects associated with BUSERELIN that are considered to be *medically important* are:

Less likely (5 – 20%):
- depression

Rarely (1 – 4%):
- visual changes
- loss of memory
- allergic reaction, in severe cases can include anaphylactic shock (anaphylaxis)
- benign tumour of the pituitary gland (pituitary adenoma)
- fever

Decreased bone density (bone loss) may occur in patients treated with long term LHRH agonists.

Thromboemboli (blood clots) have been rarely associated with the androgen suppression agents used on this trial.

During Radiation Therapy:

Very likely (5-20%):
I have been told that very likely side effects that may occur as a result of treatment with radiation therapy include:

- Diarrhea and rectal irritation which can present as a pressure or fullness in the rectum, discomfort and pain near the anus, hemorrhoid irritation and less likely bleeding.
- Bladder irritation which could cause me to pass urine more frequently (both day and night) and to have to rush to the bathroom when I get the urge to pass urine.
- Slight redness and sensitivity of skin over radiated area – I have been told that this is rarely severe.
- Fatigue and nausea (rarely vomiting) – I have been told that these are rarely severe.
- Impotence (unable to have an erection).

Following Radiation Therapy:

Rarely (1-4%):
I understand that rare severe persistent side effects of radiation therapy occur in patients and may be the following:

- Diarrhea and rectal irritation which can present as a pressure or fullness of the rectum, discomfort near the anus, hemorrhoid irritation and less likely bleeding. Very rarely a rectal ulcer may occur causing some pain, bleeding and mucous with bowel movements.
- Bladder irritation which could cause me to pass urine more frequently (both day and night) and to have to rush to the bathroom when I get the urge to pass urine.
- Impotence (unable to have an erection)
- Narrowing of the bladder neck – I understand that this could cause me to have difficulties passing urine and might make it necessary for me to have an operation.

In addition, there is always the risk of very uncommon or previously unknown side effects occurring. I understand that many of these side effects are irreversible and have been discussed by my doctor.

I understand that not enough is known about these treatments to know if they may affect an unborn child. Therefore adequate birth control measures will be used by me or my sexual partners while participating in this study.

My doctor will be watching me closely to see if side effects are occurring. My doctor may prescribe medication (or may delay the treatment) to keep these side effects under control.

If new side effects or information concerning my treatment are identified in the course of the study, I will be informed.

Potential Benefits

I have been told that it is unknown whether or not I will derive any personal benefit from participation in this study.

I understand that this study may demonstrate that hormone therapy alone is just as effective a treatment as combined hormone and radiation therapy. After many years of follow-up it may be possible to determine whether hormone therapy alone has fewer long term side effects or complications; or whether the combination of hormone therapy and radiation therapy has more side effects or complications.

I have been told that should my disease become worse, if side effects become very severe, if new information indicates that this treatment is not in my best interest, or my doctor feels that this treatment is no longer in my best interest, or the sponsor decides to stop the study, the treatment will be stopped. Further treatment would be discussed.

Alternatives

I have been told that alternatives which could be considered in my case include radiation therapy alone, other hormonal therapy, or a combination of therapies. My doctor has discussed with me the possible benefits and side effects of these other treatments.

Tests, Questionnaire

I have been told that a number of investigations (blood tests, x-rays) will be performed while I am on the study. These tests are of the same type that would be done if I was not participating in the study, but may be carried out more frequently than usual, particularly during the early stages of the study.

The needles used to take blood or inject substances for body scans might be uncomfortable. I might get a bruise, or rarely, an infection at the site of the needle puncture.

I understand that at specified intervals while on the study, I will be asked to fill out a Quality of Life questionnaire that will take 15 to 20 minutes that records how well I feel and how well I am tolerating the treatment, and that this is an important part of this study.

Confidentiality, Access to Medical Records

Qualified representatives of the following organizations may inspect and receive information from my medical/study records for quality assurance and data analysis:

- NCIC Clinical Trials Group (NCIC CTG), the research group coordinating this study
- The research ethics committee who oversees the ethical conduct of this study in your hospital/clinic
- Southwest Oncology Group
- MRC-UK
- National Cancer Institute of the U.S.
- U.S. Food and Drug Administration (because they oversee the use of drugs in the U.S.)
- Other regulatory authorities (because they oversee the use of drugs in other countries)

The organizations listed above will keep the information they see or receive about me confidential, to the extent permitted by applicable laws.

Because the long term results of this study are important, this form gives life-long consent for access to my future medical information pertaining to my cancer or treatment.

No records bearing my name will be provided to anyone other than the investigators involved in this study. I will not be identifiable as an individual in any publication which may result from this study.

Costs

I will not be paid for taking part in this study. Taking part in this study may result in added costs to me.

In the case of research-related side effects or injury, medical care will be provided by my doctor or I will be referred for appropriate medical care.

Voluntary Participation

I have discussed the information above with my doctor and she/he has answered any questions I have had about my treatment. Participation in this study is voluntary and I understand that I can refuse to participate and am free to withdraw from it at any time. If I refuse to participate, or participate and subsequently withdraw, my doctor will continue to treat me with the best means available.

I also understand that if I have questions concerning my participation in this study, I may at any time meet with the physician who is principally responsible for the project. I have received a copy of this consent form.

For patients participating through SWOG institutions:

In the event that physical injury or complications occur as a result of this research, facilities for treatment of injury will be available; however, I will not automatically be provided with reimbursement for medical care or other compensation.

For more information concerning the research and research-related risks or injuries, I can contact Dr. _______________________, the investigator in charge at (Tel)_______________.

In addition, I may contact _______________________ at (Tel)________________ for information regarding patients' rights in research studies.

The name of an individual not involved with this study with whom I may discuss issues related to my participation in this study is:

_________________________________ __________________________
Telephone

The name of the principal investigator for this project at this centre:

_________________________________ __________________________
Telephone

By signing this consent form, I am indicating that I agree to participate in this study.

_________________________________ __________________________
SIGNATURE OF PATIENT DATE

_________________________________ __________________________
SIGNATURE OF INVESTIGATOR DATE

Was the patient assisted during the consent process in one of the ways listed below?

☐ Yes ☐ No

If yes, please check the relevant box and complete the signature space below:

☐ The consent form was read to the patient, and the person signing below attests that the study was accurately explained to, and apparently understood by, the patient.

☐ *For Canadian sites only:* The person signing below acted as a translator for the patient, during the consent process.

_________________________________ __________________________
Signature of Person Assisting in the Consent Discussion Date

****

Please note that a French version of this consent form is available upon request.

APPENDIX I - PATIENT EVALUATION FLOW SHEET

Required Studies	Pre-study	Last day of radiation therapy	6 months post randomization	q 6 months x 2 years then annual until death	At relapse
<u>History and Physical including:</u>	x	x	x	x	x
performance status	x	x	x	x	x
rectal exam	x		x	x	x
<u>Laboratory</u>					
CBC, WBC diff., platelets	x	x	x	x	x
AST (SGOT)<br>ALT (SGPT) ****<br>alkaline phos.<br>total bilirubin	x		x	x	x
serum creatinine	x		x	x	x
Serum testosterone (optional)	x		x	annual	x
* PSA	x***		x	x	x
<u>Xrays and Scans</u>					
chest xray	x	if clinically indicated			
bone scan	x				
CT or MRI (of the abdomen/pelvis if req'd) (see section 6.0)	x				
<u>Other</u>					
biopsy (and local path. review for North America) †	x				x**
<u>Quality of Life</u>	x	x	x	x	x
<u>Toxicity Evaluation</u> (including libido + potency)	x	x	x	x	x

* confirmatory repeat for PSA may be required (see section 10.2.1)
** biopsy at relapse if proceeding to radiation therapy
*** if a TURP is performed, the pre randomization PSA must be obtained more than 21 days after the TURP
**** only if on long term anti-androgen therapy
† Bx & LPR at 5 years if in remission (optional)
†† last day of radiation therapy

APPENDIX II - PERFORMANCE STATUS (ECOG)

GRADE

0 Fully active, able to carry on all pre-disease performance without restriction (Karnofsky 90-100).

1 Restricted in physically strenuous activity but ambulatory and able to carry out work of a light or sedentary nature (eg, light housework, office work) (Karnofsky 70-80).

2 Ambulatory and capable of all self-care but unable to carry out any work activities. Up and about more than 50% of waking hours (Karnofsky 50-60).

3 Capable of only limited self-care, confined to bed or chair more than 50% of waking hours (Karnofsky 30-40).

4 Completely disabled. Cannot carry on any self-care. Totally confined to bed or chair (Karnofsky 10-20).

APPENDIX III - STAGING CRITERIA

DEFINITION OF TNM

Primary Tumour (T)

TX Primary tumour cannot be assessed

T0 No evidence of primary tumour

T1 Clinically inapparent tumour not palpable or visible by imaging
- T1a Tumour incidental histologic finding in 5% or less of tissue resected
- T1b Tumour incidental histologic finding in more than 5% of tissue resected
- T1c Tumour identified by needle biopsy (e.g., because of elevated PSA)

T2 Tumour confined within the prostate*
- T2a Tumour involves half of a lobe or less
- T2b Tumour involves more than half of a lobe, but not both lobes
- T2c Tumour involves both lobes

T3 Tumour extends through the prostatic capsule**
- T3a Unilateral extracapsular extension
- T3b Bilateral extracapsular extension
- T3c Tumour invades the seminal vesicle(s)

T4 Tumour is fixed or invades adjacent structures other than the seminal vesicles
- T4a Tumour invades any of: bladder neck, external sphincter, or rectum
- T4b Tumour invades levator muscles and/or is fixed to the pelvic wall

* Tumour found in one or both lobes by needle biopsy, but not palpable or visible by imaging, is classified as T1c.

** Invasion into the prostatic apex or into (but not beyond) the prostatic capsule is not classified as T3, but as T2.

APPENDIX IV - DOCUMENTATION FOR STUDY

Follow-up is required for patients from the time of randomization and will apply to all eligible patients. The follow-up for ineligible patients is minimal follow-up using a Form 5M.

Form	To Be Completed	Due in Central Office	Supporting Documentation Required
Eligibility Checklist/Form 1 Initial Evaluation	prior to calling NCIC CTG to randomize patient	within 6 weeks of randomization	* signed copy of patient consent<br>* baseline QoL questionnaire<br>* lab results (PSA)<br>* CXR, bone scan, CT/MRI reports<br>* local path. review
Form 4 Radiotherapy/Toxicity Report	after completion of radiation therapy	within 6 weeks of completion of radiation therapy	* toxicities (end of radiation therapy)<br>* lab results<br>* QoL<br>* any radiology not previously reported
Form 5 Therapy and Follow-up Report	6 months post randomization; then q 6 months x 2 years then annual until death	within 8 weeks of examination	* lab reports (PSA)<br>* QoL<br>* * radiology/pathology reports
Form 5M Follow-up Report	yearly	within 8 weeks of examination	for ineligible patients and, after trial is closed and published, for all patients
Form 6 Final Report	at time of death	within 8 weeks of death	autopsy report (if done)
Form 9 Relapse/Progressive Disease Report	at first evidence of relapse/progression	within 4 weeks of relapse/progression	* lab reports (PSA)<br>* any radiology/pathology (local review) reports documenting relapse<br>* lymph node assessment report<br>* QoL
Adverse Event Report	See section 11.0 Adverse Event Reporting for details.		
Quality of Life	accompanying Forms 1, 4, 5, 9		

SWOG Forms Submission: The original data forms as listed in Appendix IV should be submitted at the required intervals to SWOG Data Operations Center c/o Cancer Research & Biostatistics (CRAB). Include both the NCIC CTG and SWOG study numbers and patient numbers. The SWOG Data Operations Center c/o CRAB will forward the forms to the NCIC CTG. (see section 9.0)

MRC-UK Submission: see section 9.

The required forms are attached to the protocol. Additional forms packets will not be supplied when patients are randomized. It is the responsibility of the participating institution to maintain a supply of available forms for data submission.

APPENDIX V - NCIC CTG EXPANDED COMMON TOXICITY CRITERIA

Explanatory Notes

1. Toxicities are grouped into the following categories based on body system:

Allergy	Hepatic
Blood/Bone Marrow	Infection
Cancer-related Symptoms	Metabolic
Cardiovascular	Neurologic
Coagulation	Ocular
Dentition (teeth)	Osseous (bone)
Endocrine	Other
Flu-Like Symptoms	Pulmonary
Gastrointestinal	Skin
Genitourinary	Weight

2. Protocols requiring detailed hyposensitivity reaction reporting will include a Hypersensitivity Reaction Module.

3. Categories are listed alphabetically, with toxicity variables (eg. dysrhythmia, nausea, dizziness) listed alphabetically within each category.

4. Toxicity codes are composed of a 2-character "prefix" based on toxicity category, and a 3-character "description" based on variable name.

For example:	(cardiovascular) dysrhythmia	= CD DYS
	(gastrointestinal) nausea	= GI NAU
	(neurologic) dizziness	= NE DIZ

5. Some conventions:

	H = hyper (or high)	(eg. CD HBP = hypertension)
	L = HYPO (OR LOW)	(EG. MT LCA = HYPOCALCEMIA)

6. Codes are usually derived from the first 3 letters of the toxicity variable (eg. nausea = GI NAU). Exceptions to this rule have been made in the following cases:

- ➔ where the first 3 letters are not particularly helpful or descriptive (eg. mouth dryness has been coded GI DRY instead of GI MOU)
- ➔ where the first 3 letters are potentially confusing (eg. flushing, facial has been coded SK FAC instead of SK FLU)
- ➔ where a "common" 3 letter abbreviation already exists (eg. hemoglobin has been coded BL HGB instead of BL HEM)

7. For toxicities which do not have an existing code, but do fit into an existing toxicity category, use "other" variable in the appropriate toxicity category (eg. code pathologic fracture OSSEOUS OTHER (OS OTH)). For toxicities which do not have existing codes, and do NOT fit into existing categories, code OTHER OTHER (OT OTH).

8. Please note that ONLY the codes listed in the criteria may be used. Data managers should not "create" new toxicity codes. If a new toxicity is identified which doesn't have an existing code or doesn't fit an existing category, use OTHER and OTHER OTHER variables as outlined above. If you're unsure how to code a particular toxicity, please record toxicity type only on the form. A coding decision will then be made at the NCIC CTG central office.

Explanatory Notes Revised 94-Dec-21

NCIC CTG EXPANDED COMMON TOXICITY CRITERIA					REVISED: 94-DEC-21
Grade	0	1	2	3	4
ALLERGY					
AL LER Allergy	none	transient rash, fever < 38°C, 100.4°F	urticaria, fever=38°C, 100.4°F, mild bronchospasm	serum sickness, bronchospasm, req parenteral meds	anaphylaxis
	Fever felt to be caused by drug allergy should be coded as ALLERGY (AL LER). Non-allergic drug fever (eg. as from biologics) should be coded under FLU-LIKE SYMPTOMS (FL FEV). If fever is due to infection, code INFECTION only (IN FEC or IN NEU). NB: Protocols requiring detailed reporting of hypersensitivity reactions, will include a Hypersensitivity Reaction module.				
AL OTH Other*	none	mild	moderate	severe	life threatening
BLOOD/BONE MARROW (SI UNITS)					
BL WBC White Blood Count (WBC)	≥4.0 10^9/L	3.0-3.9	2.0-2.9	1.0-1.9	<1.0
BL PLT Platelets	WNL 10^9/L	75.0-normal	50.0-74.9	25.0-49.9	<25.0
BL HGB Hemoglobin (Hgb)	WNL g/L	100-normal	80-99	65-79	<65
BL GRA Granulocytes (i.e. neuts + bands)	≥2.0 10^9/L	1.5-1.9	1.0-1.4	0.5-0.9	<0.5
BL LYM Lymphocytes	≥2.0 10^9/L	1.5-1.9	1.0-1.4	0.5-0.9	<0.5
BL HEM Hemorrhage resulting from thrombocytopenia (clinical)	none	mild, no transfusion (incl bruise/hematoma, petechiae)	gross, 1-2 units transfusion per episode	gross, 3-4 units transfusion per episode	massive, >4 units transfusion per episode
BL OTH Other*	none	mild	moderate	severe	life threatening
CANCER RELATED SYMPTOMS					
CA DEA Death from malignant disease within 30 days of treatment* (grade=5)	--	--	--	--	--
CA PAI Cancer pain*	none	pain, but no treatment req	pain controlled with non-opioids	pain controlled with opioids	uncontrollable pain
CA SEC Second malignancy*	none	--	--	present	--
CA OTH Other*	none	mild	moderate	severe	life threatening
CARDIOVASCULAR					
CD ART Arterial* (non myocardial)	none	--	--	transient events (eg. transient ischemic attack)	permanent event (eg. cerebral vascular accident)
CD VEN Venous*	none	superficial (excl IV site reaction→code SK LTO)	deep vein thrombosis not req anticoagulant therapy	deep vein thrombosis req anticoagulant therapy	pulmonary embolism
CD DYS Dysrhythmias	none	asymptomatic, transient, req no therapy	recurrent or persistent, no therapy req	req trt	req monitoring, or hypotension, or ventricular tachy-cardia, or fibrillation
CD EDE Edema* (eg. peripheral edema)	none	1+ or dependent in evening only	2+ or dependent throughout day	3+	4+, generalized anasarca
CD FUN Function	none	asymptomatic, decline of resting ejection fraction of ≥ 10% but < 20% of baseline value	asymptomatic, decline of resting ejection fraction by >20% of baseline value	mild CHF, responsive to therapy	severe or refractory CHF
CD HBP Hypertension	none or no change	asymptomatic, transient increase by >20mm Hg (D) or to >150/100 if previously WNL. No trt req	recurrent or persistent increase by >20mm Hg (D) or to >150/100 if previously WNL. No trt req	req therapy	hypertensive crisis
CD LBP Hypotension	none or no change	changes req no therapy (incl transient orthostatic hypotension)	req fluid replacement or other therapy but not hospitalization	req therapy & hospitalization; resolves within 48hrs of stopping agent	req therapy & hospitalization for >48hrs after stopping agent

NCIC CTG EXPANDED COMMON TOXICITY CRITERIA					REVISED: 94-DEC-21
Grade	0	1	2	3	4
CD ISC Ischemia (myocardial)	none	non-specific T wave flattening	asymptomatic, ST & T wave changes suggesting ischemia	angina without evidence for infarction	acute myocardial infarction
CD PAI Pain (chest)*	none	pain, but no treatment req	pain controlled with non-opioids	pain controlled with opioids	uncontrollable pain
CD PER Pericardial	none	asymptomatic, effusion, no intervention req	pericarditis (rub, chest pain, ECG changes)	symptomatic effusion; drainage req	tamponade, drainage urgently req; or constrictive pericarditis req surgery
CD TAC Sinus tachycardia*	none	mild	moderate	severe	life threatening
CD OTH Other*	none	mild	moderate	severe	life threatening
COAGULATION					
CG FIB Fibrinogen	WNL	0.99-0.75 x N	0.74-0.50 x N	0.49-0.25 x N	≤0.24 x N
CG PT Prothrombin time	WNL	1.01-1.25 x N	1.26-1.50 x N	1.51-2.00 x N	>2.00 x N
CG PTT Partial thromboplastin time	WNL	1.01-1.66 x N	1.67-2.33 x N	2.34-3.00 x N	>3.00 x N
CG OTH Other*	none	mild	moderate	severe	life threatening
DENTITION (TEETH)					
DE DEC Tooth decay*	none	mild	moderate	severe	-
DE PAI Toothache*	none	pain, but no treatment req	pain controlled with non-opioids	pain controlled with opioids	uncontrollable pain
DE OTH Other*	none	mild	moderate	severe	life threatening
ENDOCRINE *					
EN AME Amenorrhea	none	irregular menses	≥ 3 mths	--	--
EN CUS Cushingoid	normal	mild	pronounced	--	--
EN FLA Hot flashes	none	mild or <1/day	moderate & ≥1/day	frequent & interferes with normal function	--
EN GYN Gynecomastia	normal	mild	pronounced or painful	--	--
EN IMP Impotence/ Libido	normal	decrease in normal function	--	absence of function	--
EN OTH Other	none	mild	moderate	severe	life threatening
FLU-LIKE SYMPTOMS					
FL FEV Fever in absence of infection* (incl drug fever)	none	37.1-38.0°C 98.7-100.4°F	38.1-40.0°C 100.5-104.0°F	>40.0°C >104.0°F for <24hrs	>40.0°C (104.0°F) for >24hrs or fever accompanied by hypotension
Fever felt to be caused by drug allergy should be coded as ALLERGY (AL LER). Non-allergic drug fever (eg. as from biologics) should be coded under FLU-LIKE SYMPTOMS (FL FEV). If fever is due to infection, code INFECTION only (IN FEC or IN NEU).					
FL HAY Hayfever* (incl sneezing, nasal stuffiness, post-nasal drip)	none	mild	moderate	severe	--
FL JOI Arthralgia* (joint pain)	none	mild	moderate	severe	--
FL LET Lethargy* (fatigue, malaise)	none	mild, or fall of 1 level in performance status	moderate, or fall of 2 levels in perf. status	severe, or fall of 3 levels in perf. status	--
FL MYA Myalgia* (muscle ache)	none	mild	moderate	severe	--
FL RIG Rigors/Chills* (Gr 3 incl cyanosis)	none	mild or brief	pronounced and/or prolonged	cyanosis	--
FL SWE Sweating* (diaphoresis)	none	mild	moderate	severe	--
FL OTH Other*	none	mild	moderate	severe	life threatening
GASTROINTESTINAL					
GI ANO Anorexia*	none	mild	moderate	severe	dehydration
GI APP Appetite--increased*	none	mild	moderate	--	--

*denotes NCIC CTG specific criteria Any toxicity which causes death should be given a grade 5.

NCIC CTG Expanded Common Toxicity Criteria					Revised: 94-Dec-21
Grade	0	1	2	3	4
GI ASC Ascites (non-malignant)*	none	mild	moderate	severe	life threatening
GI DIA Diarrhea	none	increase of 2-3 stools/day; or mild increase in loose watery colostomy output compared to pre-trt	increase of 4-6 stools/day, or nocturnal stools; or moderate increase in loose watery colostomy output compared to pre-trt	increase of 7-9 stools/day, or incon-tinence, malabsorption; or severe increase in loose watery colostomy output compared to pre-trt	increase of ≥10 stools/day or grossly bloody diarrhea; or grossly bloody colostomy output or loose watery colostomy output req parenteral support; dehydration
GI DPH Esophagitis/ dysphagia/ odynophagia* (incl recall reaction)	none	dys. or odyn. not req trt, or painless ulcers on esophagoscopy	dys. or odyn. req trt	dys. or odyn. lasting >14 days despite trt	dys. or odyn. with 10% loss of body wt, dehydration, hosp. req
GI DRY Mouth, nose dryness*	none	mild	moderate	severe	--
GI FIS Fistula (intestinal, esophageal, rectal)*	none	--	--	req intervention	req operation
GI GAS Flatulence*	none	mild	moderate	severe	--
GI HEA Heartburn* (incl dyspepsia)	none	mild	moderate	severe	--
GI HEM Gastrointestinal bleeding*	none	mild, no transfusion	gross, 1-2 units transfusion per episode	gross, 3-4 units transfusion per episode	massive, >4 units transfusion per episode
	Bleeding resulting from thrombocytopenia should be coded under BL HEM, not GI				
GI NAU Nausea	none	able to eat reasonable intake	intake significantly decreased but can eat	no significant intake	--
GI OBS Small bowel obstruction*	none	--	intermittent, no intervention	req intervention	req operation
GI PAI Gastrointestinal pain/cramping* (incl rectal pain)	none	pain, but no treatment req	pain controlled with non-opioids	pain controlled with opioids	uncontrollable pain
GI PRO Proctitis (rectal)	none	perianal itch, hemorrhoids	tenesmus or ulcerations relieved with therapy, anal fissure	tenesmus or ulcerations or other symptoms not relieved with therapy	mucosal necrosis with hemorrhage or other life threatening proctitis
GI STO Stomatitis/oral	none	painless ulcers, erythema, or mild soreness	painful erythema, edema, or ulcers but can eat	painful erythema, edema, or ulcers, and cannot eat	mucosal necrosis and/or req parenteral or enteral support, dehydration
GI TAS Taste, sense of smell altered*	none	mild	moderate	severe	--
GI ULC Gastritis/ulcer*	none	antacid	req vigorous medical management or non-surgical trt	uncontrolled by medical management; req surgery for GI ulceration	perforation or bleeding
GI VOM Vomiting	none	1 episode in 24hrs	2-5 episodes in 24hrs	6-10 episodes in 24hrs	>10 episodes in 24hrs or req parenteral support, dehydration
GI OTH Other*	none	mild	moderate	severe	life threatening
Genitourinary					
GU BLA Bladder changes*	none	light epithelial atrophy, or minor telangiectasia	generalized telangiectasia	severe generalized telangiectasia (often with petechiae) or reduction in bladder capacity (<15ml)	necrosis, or contracted bladder (capacity <100ml), or fibrosis
GU CRE Creatinine	WNL	<1.5 x N	1.5-3.0 x N	3.1-6.0 x N	>6.0 x N
GU CYS Cystitis* (non-bacterial)	none	mild symptoms req no intervention	symptoms relieved completely with therapy	symptoms not relieved despite therapy	severe (life threatening) cystitis
	Urinary tract infection should be coded under infection, not GU.				

*denotes NCIC CTG specific criteria   Any toxicity which causes death should be given a grade 5.

NCIC CTG EXPANDED COMMON TOXICITY CRITERIA							REVISED: 94-DEC-21
		Grade	0	1	2	3	4
GU	FIS	Fistula* (vaginal, vesicovaginal)	none	--	--	req intervention	req operation
GU	FRE	Frequency*	none	freq of urination or nocturia twice pre-trt habit	freq of urination or nocturia <hourly	freq with urgency and nocturia ≥hourly	--
GU	HEM	Hematuria, bleeding per vagina	neg	micro only	gross, no clots	gross + clots	req transfusion
			Bleeding resulting from thrombocytopenia should be coded under BL HEM not GU.				
GU	INC	Incontinence*	none	mild	moderate	severe	--
GU	OBS	Ureteral obstruction*	none	unilateral, no surgery	bilateral, no surgery req	not complete bilateral, but stents, nephrostomy tubes or surgery req	complete bilateral obstruction
GU	PAI	Genito-urinary pain* (eg: dysuria, dysmenorrhea, dyspareunia)	none	pain, but no treatment req	pain controlled with non-opioids	pain controlled with opioids	uncontrollable pain
GU	PRT	Proteinuria	no change	1+<br>or <0.3 g%<br>or <3g/L	2-3+<br>or 0.3-1.0g%<br>or 3-10g/L	4+<br>or >1.0g%<br>or >10g/L	nephrotic syndrome
GU	VAG	Vaginitis* (+/- vaginal discharge) (non-infectious)	none	mild, no trt req	moderate, relieved with trt	severe, not relieved with trt	life threatening
GU	OTH	Other*	none	mild	moderate	severe	life threatening
Hepatic							
HP	ALK	Alk Phos or 5'nucleotidase	WNL	≤2.5 x N	2.6-5.0 x N	5.1-20.0 x N	>20.0 x N
HP	ALT	Transaminase SGPT (ALT)	WNL	≤2.5 x N	2.6-5.0 x N	5.1-20.0 x N	>20.0 x N
HP	AST	Transaminase SGOT (AST)	WNL	≤2.5 x N	2.6-5.0 x N	5.1-20.0 x N	>20.0 x N
HP	BIL	Bilirubin	WNL	--	<1.5 x N	1.5-3.0 x N	>3.0 x N
HP	CLI	Liver (clinical)	no change from baseline	--	--	precoma	hepatic coma
HP	LDH	LDH*	WNL	<2.5 x N	2.6-5.0 x N	5.1-20.0 x N	>20.0 x N
HP	OTH	Other*	none	mild	moderate	severe	life threatening
			Viral Hepatitis should be coded as infection rather than liver toxicity.				
Infection							
IN	FEC	Infection	none	mild, no active trt	moderate, localized infect req active trt	severe, systemic infect req parenteral trt, specify site	life threatening sepsis, specify site
IN	NEU	Febrile neutropenia* Absolute gran. count $<1.0x10^9$/L, fever ≥38.5°C treated with (or ought to have been treated with) IV antibiotics	none	--	--	present	--
			Fever felt to be caused by <u>drug allergy</u> should be coded as ALLERGY (AL LER). <u>Non-allergic</u> drug fever (eg. as from biologics) should be coded under FLU-LIKE SYMPTOMS (FL FEV). If fever is due to <u>infection</u>, code INFECTION only (IN FEC <u>or</u> IN NEU).				
Metabolic (SI units)							
MT	AMY	Amylase	WNL	<1.5 x N	1.5-2.0 x N	2.1-5.0 x N	>5.1 x N
MT	HCA	Hypercalcemia	<2.64 mmol/L	2.64-2.88	2.89-3.12	3.13-3.37	≥3.37
MT	LCA	Hypocalcemia	>2.10 mmol/L	2.10-1.93	1.92-1.74	1.73-1.51	≤1.50
MT	HGL	Hyperglycemia	<6.44 mmol/L	6.44-8.90	8.91-13.8	13.9-27.8	>27.8 or ketoacidosis
MT	LGL	Hypoglycemia	>3.55 mmol/L	3.03-3.55	2.19-3.02	1.66-2.18	<1.66
MT	LKA	Hypokalemia*	no change or >3.5 mmol/L	3.1-3.5	2.6-3.0	2.1-2.5	≤2.0
MT	LMA	Hypomagnesemia	>0.70 mmol/L	0.70-0.58	0.57-0.38	0.37-0.30	≤0.29
MT	LNA	Hyponatremia*	no change or >135 mmol/L	131-135	126-130	121-125	≤120

*denotes NCIC CTG specific criteria   Any toxicity which causes death should be given a grade 5.

NCIC CTG Expanded Common Toxicity Criteria					Revised: 94-Dec-21
Grade	0	1	2	3	4
MT OTH Other*	none	mild	moderate	severe	life threatening
**Neurologic**					
NE CER Cerebellar	none	slight incoordination, dysdiadochokinesis	intention tremor, dysmetria, slurred speech, nystagmus	locomotor ataxia	cerebellar necrosis
NE CON Constipation	none or no change	mild	moderate	severe, obstipation	ileus >96hrs
NE COR Cortical (incl drowsiness)	none	mild somnolence	moderate somnolence	severe somnolence, confusion, disorienta-tion, hallucinations	coma, seizures, toxic psychosis
NE DIZ Dizziness* (incl lightheadedness)	none	mild	moderate	severe (incl fainting)	--
NE EXT Extrapyramidal/ Involuntary movement*	none	mild agitation (incl restlessness)	moderate agitation	torticollis, oculogyric crisis, severe agitation	--
NE HED Headache	none	mild	moderate or severe but transient	unrelenting & severe	--
NE HER Altered hearing	none or no change	asymptomatic, hearing changes on audiometry only	tinnitus, symptomatic hearing changes not req hearing aid or trt	hearing changes inter-fering with function but correctable with hearing aid or trt	hearing changes or deafness not correctable
NE INS Insomnia*	none	mild	moderate	severe	--
NE MOO Mood	no change	mild anxiety or depression	moderate anxiety or depression	severe anxiety or depression	suicidal ideation
NE MOT Motor	none or no change	subjective weakness; no objective findings	mild objective weakness without significant impairment of function	objective weakness with impairment of function	paralysis
NE PAI Neurologic pain* (eg: jaw pain)	none	pain, but no treatment req	pain controlled with non-opioids	pain controlled with opioids	uncontrollable pain
NE PER Personality Change*	no change	change, not disruptive to pt or family	disruptive to pt or family	harmful to others or self	psychosis
NE SEN Sensory	none or no change	mild paresthesias, loss of deep tendon reflexes (incl tingling)	mild or moderate objective sensory loss; moderate paresthesias	sensory loss or paresthesias that interfere with function	--
NE VIS Vision	none or no change	blurred vision	--	symptomatic subtotal loss of vision	blindness
NE OTH Other*	none	mild	moderate	severe	life threatening
**Ocular**					
OC CAT Cataract*	none	mild	moderate	severe	--
OC CJN Conjunctivitis/ Keratitis	none	erythema or chemosis not req steroids or antibiotics	req trt with steroids or antibiotics	corneal ulceration or visible opacification	--
OC DRY Dry eye	normal	mild	req artificial tears	severe	req enucleation
OC GLA Glaucoma	no change	--	--	yes	--
OC PAI Eye pain*	none	pain, but no treatment req	pain controlled with non-opioids	pain controlled with opioids	uncontrollable pain
OC TEA Tearing* (watery eyes)	none	mild	moderate	severe	--
OC OTH Other	none	mild	moderate	severe	life threatening
**Osseous (bone)**					
OS PAI Bone pain*	none	pain, but no treatment req	pain controlled with non-opioids	pain controlled with opioids	uncontrollable pain
OS OTH Other* (eg: avascular necrosis)	none	mild	moderate	severe	life threatening
**Other**					
OT OTH Other	none	mild	moderate	severe	life threatening
	For toxicities which do not have an existing code, but do fit into an existing toxicity category, use "other" variable in the appro-priate toxicity category. Only toxicities which do not fit into existing categories should be coded OTHER OTHER (OT OTH).				

*denotes NCIC CTG specific criteria Any toxicity which causes death should be given a grade 5.

NCIC CTG EXPANDED COMMON TOXICITY CRITERIA							REVISED: 94-DEC-21
		Grade	0	1	2	3	4
Pulmonary							
PU	CMD	Carbon Monoxide Diffusion Capacity (DLCO)*	>90% of pretreatment value	decrease to 76-90% of pre-trt	decrease to 51-75% of pre-trt	decrease to 26-50% of pre-trt	decrease to ≤25% of pre-trt
PU	COU	Cough*	none	mild	moderate	severe	--
PU	EDE	Pulmonary Edema*	none	--	out-pt management	in-pt management	req intubation
PU	EFF	Pleural effusion* (non-malignant)	none	mild	moderate	severe	life threatening
PU	FIB	Pulmonary Fibrosis*	normal	radiographic changes, no symptoms	--	changes with symptoms	--
PU	HEM	Hemoptysis*	none	mild, no transfusion	gross, 1-2 units transfusion per episode	gross, 3-4 units transfusion per episode	massive, >4 units transfusion per episode
			Bleeding resulting from thrombocytopenia should be coded under BL HEM, not PU				
PU	HIC	Hiccoughs*	none	mild	moderate	severe	--
PU	PAI	Pulmonary pain*	none	pain, but no treatment req	pain controlled with non-opioids	pain controlled with opioids	uncontrollable pain
PU	PNE	Pneumonitis* (non-infectious)	normal	radiographic changes, symptoms do not req steroids	steroids req	oxygen req	req assisted ventilation
PU	SOB	Shortness of breath (SOB) (incl wheezing)	none or no change	asymptomatic, with abnormality in PFT's	dyspnea on significant exertion	dyspnea at normal level of activity, apnea without cyanosis	dyspnea at rest, apnea with cyanosis
PU	VOI	Voice changes* (incl hoarseness, loss of voice)	none	mild	moderate	severe	--
PU	OTH	Other*	none	mild	moderate	severe	life threatening
			Pneumonia is considered infection and not graded as pulmonary toxicity unless felt to be resultant from pulmonary changes directly induced by treatment.				
Skin							
SK	ALO	Alopecia	no loss	mild hair loss	pronounced or total head hair loss	total body hair loss	--
SK	CHA	Skin changes* (eg: photosensitivity)	none	localized pigmentation changes	generalized pigmenta-tion changes or atrophy	subcut. fibrosis or localized shallow ulceration	generalized ulcerations or necrosis
SK	DES	Desquamation*	none	dry desquamation	moist desquamation	confluent moist desquamation	--
SK	DRY	Dry skin*	none	mild	moderate	severe	--
SK	FAC	Flushing* (eg: facial)	none	mild	moderate	severe	--
SK	HEM	Bruising/bleeding	none	mild, no transfusion	gross, 1-2 units transfusion per episode	gross, 3-4 units transfusion per episode	massive, >4 units transfusion per episode
			Bleeding resulting from thrombocytopenia should be coded under BL HEM, not SK				
SK	LTO	Local Toxicity (reaction at IV site)	none	pain	pain & swelling, with inflammation or phlebitis	ulceration	plastic surgery indicated
SK	NAI	Nail changes*	none	mild	moderate	severe	--
SK	PAI	Skin pain* (incl scalp pain)	none	pain, but no treatment req	pain controlled with non-opioids	pain controlled with opioids	uncontrollable pain
SK	RAS	Rash/Itch* (not due to allergy) (incl recall reaction)	none or no change	scattered macular or papular eruption or erythema that is asymptomatic	scattered macular or papular eruption or erythema with pruritus or other associated symptoms	generalized symptomatic macular, papular, or vesicular eruption	exfoliative dermatitis or ulcerating dermatitis
SK	OTH	Other*	none	mild	moderate	severe	life threatening
Weight							
WT	GAI	Weight Gain	<5.0%	5.0-9.9%	10.0-19.9%	≥20.0%	--
WT	LOS	Weight Loss	<5.0%	5.0-9.9%	10.0-19.9%	≥20.0%	--

*denotes NCIC CTG specific criteria Any toxicity which causes death should be given a grade 5.

APPENDIX VI - LIST OF "CONTACTS"

PATIENT RANDOMIZATION: All patients MUST be registered by telephone with NCIC CTG.			
	Contact	Tel. #	Fax #
ELIGIBILITY CHECKLIST<br><br>MUST be completed prior to the telephone call to request an allocation	Sue Steacy<br>Clinical Trials Assistant<br>NCIC CTG<br><br>E-Mail:<br>ssteacy@ctg.queensu.ca	(613) 533-6430	(613) 533-2941
STUDY SUPPLIES<br>Forms, Protocols			
GENERAL PROTOCOL-RELATED QUERIES	Andrea Hiltz<br>Study Coordinator<br>NCIC CTG<br><br>E-Mail:<br>ahiltz@ctg.queensu.ca		
	OR: Dr. Wendy Parulekar<br>Physician Coordinator<br>NCIC CTG<br>E-Mail:<br>wparulekar@ctg.queensu.ca		
	OR: Dr. Padraig Warde<br>Study Chair<br>E-Mail: padraig_warde@rmp.uhn.on.ca	(416) 946-2122	(416) 946-4586
	OR: Dr. Edmund Kostashuk<br>Study Co-Chair	(604) 877-6000	(604) 872-4596
	OR: Dr. Gregory P. Swanson<br>SWOG Study Co-chair	(509) 455-5930	(509) 455-4023
	OR: Dr. Malcolm Mason<br>MRC-UK Study Co-chair	+44 (0) 1222<br>615 888	+44 (0) 1222<br>529 625
	OR: Mr. James Latham<br>MRC UK Prostate Cancer Manager	+44 (0) 2076<br>704-831	+44 (0) 2076<br>704-818
ADVERSE EVENT REPORTING<br><br>See protocol Section 11.0 for details of reportable events	Andrea Hiltz<br>Study Coordinator<br>NCIC CTG	(613) 533-6430	(613) 533-2941

APPENDIX VII - QUALITY OF LIFE ASSESSMENT

Instructions for Data Managers (CRA)

Each North American centre will be assigned to use the QLQ-C30+3 and trial specific checklist (PR17) or the FACT-P instrument. Centres will receive questionnaires of only that version assigned.

Patients must be willing and able to complete the quality of life questionnaires in either English or French. Should there be sufficient reason why a patient cannot complete these assessments (illiteracy, loss of sight or other equivalent reasons) this inability will not make the patient ineligible for the study nor inevaluable for the main objective of the study.

The brief instructions given below are intended as a guide for the quality of life (QoL) questionnaire.

1. The QoL assessment is in the form of a self-report questionnaire. Therefore, it must be completed by the patient only, without coaching or suggestions as to the "correct" answer by relatives or health care personnel.

2. The CRA should give the questionnaire directly to the patient before the patient is seen by the physician at the clinic visits indicated in the protocol. (Generally, there is a brief waiting period to see the physician and this is a good time to fill out the questionnaire, since it only takes 10-15 minutes to complete).

3. The CRA should explain that the purpose of the questionnaire is to obtain information which is additional to the information usually gathered at clinic visits and state that the CRA will be in the vicinity should the patient have questions about the questionnaire. The CRA may provide clarification, but should not rephrase the questions, suggest answers or discuss the answers. (An explanation of the purpose will likely be required on the first occasion that the questionnaire is presented).

4. The CRA should collect the questionnaire as soon as it has been completed, check the answers to see that each question has been answered and gently remind the patient to answer any questions that may not have been answered in case they were omitted inadvertently. If the patient states that he/she prefers not to answer some questions despite a reminder from the CRA, the CRA should not encourage the patient to provide an answer, but subsequently should note "prefers not to answer questions ___" and sign at the end of the questionnaire.

5. Each completed questionnaire should be sent within seven days of completion to the coordinating group office.

6. Questionnaires should not be mailed to patients, except in very exceptional circumstances such as:

   (a) failure to give the questionnaire to the patient during a clinic visit,

   (b) when a patient decides not to keep an appointment for follow-up and is unlikely to return for future follow-up because of:

      (i) a wish to go off study,
      (ii) move to another locale where the patient will not be followed on study,
      (iii) recurrent cancer and unlikely to return to clinic, or
      (iv) some similar reason.

If one of these circumstances were to occur, the completed questionnaire should be accompanied by a note from the CRA explaining why the questionnaire was mailed to the patient.

7. The patient should be encouraged to complete questionnaire in clinic, preferably before seeing the physician, and should not take the questionnaire home for return (in person or by mail) at a later time (except as noted in 6 above). (Varying the environment in which the questionnaire is completed by allowing completion at times other than at the time of the clinic visit introduces unnecessary variables into the study). Should the patient insist on taking the questionnaire home, and failure to comply with the patient's wishes is likely to result in the questionnaire not being completed at all, then the patient may take the questionnaire home with the instructions that it is to be completed the same day. When the questionnaire is returned, the CRA should check the date on which the questionnaire was completed and attach a note to it stating why the patient took it away from the clinic before completion.

8. The information provided by the patient in the completed questionnaire is confidential and should not be discussed with or shown to anyone who is not mentioned in the consent form signed by the patient.

SCHEDULE FOR QUALITY OF LIFE ASSESSMENTS

Pre randomization	All patients to complete quality of life assessment within 28 days prior to randomization.
Radiation therapy	Those patients assigned to radiation therapy should complete the quality of life assessment on the last day of that therapy.
Follow-up	All patients should complete the quality of life assessment at 6 months post randomization and every 6 months x 2 years and then annually thereafter until death.
At relapse	All patients should complete the quality of life assessment at time of relapse.

Intergroup (NCIC CTG, CUOG, ECOG, CALGB and SWOG) Phase III Randomized Trial Comparing Total Androgen Blockade Versus Total Androgen Blockade Plus Pelvic Irradiation in Clinical Stage T3-4, N0, M0 Adenocarcinoma of the Prostate

Quality of Life Questionnaire

Instructions: This cover sheet is to be completed by nurse/clinical research associate/investigator.

This cover sheet must be completed for all patients whether the questionnaire is completed or not by the patient.

Attach the completed cover sheet to the Questionnaire when returned by the patient.

Note: If the entire questionnaire is not completed, please complete and return this cover page with the case report form.

Patient NCIC CTG Serial #: ____________ Patient Hospital #: _________________ Patient Initials: ____ ____ ____ (first, middle, last)

Intergroup Patient Serial #: _____________ (U.S. Patients: Soc. Sec. #: _______________________)

Institution: ____ Investigator: ____________________________________

Scheduled time to obtain quality of life assessment: please check (3)

- ☐ Within 28 days prior to randomization
- ☐ last day of radiation therapy (Arm B only)
- ☐ 6 months post randomization
- ☐ every 6 months x 2 years:
- ☐ annually:
- ☐ at relapse

Month of Report

☐ 12 ☐ 18 ☐ 24

☐ 36 ☐ 48 ☐ 60 ☐ specify_____

Was questionnaire completed? ___ Yes ➤ Date questionnaire completed: __ __ - __ __ __ - __ __ (yy mmm dd)

___ No ➤ Please fill in today's date: __ __ - __ __ __ - __ __ (yy mmm dd)

Specify reason questionnaire not completed (check one):

___ 1. Patient kept appointment for examination, but could not complete Questionnaire due to illness.

___ 2. Patient kept appointment for examination, but refused to complete Questionnaire for reason other than illness.
Specify reason: ____

___ 3. Patient did not keep appointment.
Specify reason: ____

___ 4. Patient could not be contacted.

___ 5. Questionnaire not administered due to institution error.

___ 6. Other reason, specify:

Were ALL questions answered? ___ Yes ___ No If no, reason: ____

Was assistance required? ___ Yes ___ No If yes, reason: ____

NCIC CTG use only

Logged: _______	Study Coord:______	Phy: ______	Data Ent'd:	Verif:
___ - ____ - ___	___ - ____ - ___	___ - ____ - ___	______	______

This box to be completed by the clinic nurse or clinical research associate: Pt. Serial #: __________ Pt. Initials: ___ ___ ___

European Organization for Research and Treatment of Cancer (EORTC)

Quality of Life Questionnaire (PR.3)

We are interested in some things about you and your health. Please answer all the questions yourself by circling the number that best applies to you. There are no 'right' or 'wrong' answers. The information that you provide will remain strictly confidential.

		No	Yes
1.	Do you have any trouble doing strenuous activities, like carrying a heavy shopping bag or a suitcase?	1	2
2.	Do you have any trouble taking a long walk?	1	2
3.	Do you have any trouble taking a short walk outside of the house?	1	2
4.	Do you have to stay in a bed or a chair for most of the day?	1	2
5.	Do you need help with eating, dressing, washing yourself or using the toilet?	1	2
6.	Are you limited in any way in doing either your work or doing household jobs?	1	2
7.	Are you completely unable to work at a job or to do household jobs?	1	2

During the past week:		Not at All	A Little	Quite a Bit	Very Much
8.	Were you short of breath?	1	2	3	4
9.	Have you had pain?	1	2	3	4
10.	Did you need to rest?	1	2	3	4
11.	Have you had trouble sleeping?	1	2	3	4
12.	Have you felt weak?	1	2	3	4
13.	Have you lacked appetite?	1	2	3	4
14.	Have you felt nauseated?	1	2	3	4

Please go on to the next page
EORTC QOL Core 30+3 (Core 30 Questionnaire 8 copyright 1992 EORTC Study Group on Quality of Life. All rights reserved.)

This box to be completed by the clinic nurse or clinical research associate: Pt. Serial #: __________ Pt. Initials: ___ ___ ___

During the past week:	Not at All	A Little	Quite a Bit	Very Much
15. Have you vomited?	1	2	3	4
16. Have you been constipated?	1	2	3	4
17. Have you had diarrhea?	1	2	3	4
18. Were you tired?	1	2	3	4
19. Did pain interfere with your daily activities?	1	2	3	4
20. Have you had difficulty in concentrating on things like reading a newspaper or watching television?	1	2	3	4
21. Did you feel tense?	1	2	3	4
22. Did you worry?	1	2	3	4
23. Did you feel irritable?	1	2	3	4
24. Did you feel depressed?	1	2	3	4
25. Have you had difficulty remembering things?	1	2	3	4
26. Are you limited in doing either your work or household jobs?	1	2	3	4
27. Are you limited in pursuing your hobbies or other leisure time activities?	1	2	3	4
28. Has your physical condition or medical treatment interfered with your family life?	1	2	3	4
29. Has your physical condition or medical treatment interfered with your social activities?	1	2	3	4
30. Has your physical condition or medical treatment caused you financial difficulties?	1	2	3	4

Please go on to the next page

EORTC QOL Core 30+3 (Core 30 Questionnaire 8 copyright 1992 EORTC Study Group on Quality of Life. All rights reserved.)

This box to be completed by the clinic nurse or clinical research associate: Pt. Serial #: __________ Pt. Initials: ___ ___ ___

For the following questions please circle the number between 1 and 7 that best applies to you.

31. How would you rate your overall physical condition during the past week?

1	2	3	4	5	6	7
Very Poor						Excellent

32. How would you rate your overall health during the past week?

1	2	3	4	5	6	7
Very Poor						Excellent

33. How would you rate your overall quality of life during the past week?

1	2	3	4	5	6	7
Very Poor						Excellent

Patients sometimes report that they have the following symptoms. Please indicate the extent to which you have experienced these symptoms during the past week, by circling the number that best applies to you.

During the past week:	Not at All	A Little	Quite a Bit	Very Much
34. Did you have to pass urine more frequently than normal for you?	1	2	3	4
35. Did you have difficulty passing your urine?	1	2	3	4
36. Did you have pain when you passed urine?	1	2	3	4
37. Did you have blood in your urine?	1	2	3	4
38. Did you have difficulty emptying your bladder completely?	1	2	3	4
39. Did you have difficulty controlling your urination (for example dribbling)?	1	2	3	4
40. Did you have accidental wetting of your underwear?	1	2	3	4
41. Did you have to wear added protection to prevent accidental wetting of your underwear?	1	2	3	4

 Please go on to the next page

This box to be completed by the clinic nurse or clinical research associate: Pt. Serial #: __________ Pt. Initials: ___ ___ ___

During the past week:	Not at All	A Little	Quite a Bit	Very Much
42. Did you have any bleeding from your rectum (for example, with a bowel movement)?	1	2	3	4
43. Did you have any pain in your rectum?	1	2	3	4
44. Did you have hot flashes?	1	2	3	4
45. Did you have any bothersome breast enlargement?	1	2	3	4
46. Has your present condition affected your sex life?	1	2	3	4
47. Did you limit your activities outside your home?	1	2	3	4
If you answered "not at all", please proceed to question 49.				
48. If you limited your activities outside the home, was this because of your urination problems?	1	2	3	4
49. Did you have to get up at night to pass urine?	1	2	3	4
If you answered "not at all", please proceed to next page.				
50. How much did getting up at night interfere with your sleep?	1	2	3	4

Please go on to the next page

We would like to know if there have been any changes since the last time you filled out the questionnaire. Please circle your answer.

1. Since the last time I filled out the questionnaire, my physical condition is:

1	2	3	4	5	6	7
Very much worse	Moderately worse	A little worse	About the same	A little better	Moderately better	Very much better

2. Since the last time I filled out the questionnaire, my emotional state is:

1	2	3	4	5	6	7
Very much worse	Moderately worse	A little worse	About the same	A little better	Moderately better	Very much better

3. Since the last time I filled out the questionnaire, my ability to enjoy social life is:

1	2	3	4	5	6	7
Very much worse	Moderately worse	A little worse	About the same	A little better	Moderately better	Very much better

4. Since the last time I filled out the questionnaire, my symptoms in general...that is, overall physical comfort is:

1	2	3	4	5	6	7
Very much worse	Moderately worse	A little worse	About the same	A little better	Moderately better	Very much better

5. Since the last time I filled out the questionnaire, the overall quality of my life is:

1	2	3	4	5	6	7
Very much worse	Moderately worse	A little worse	About the same	A little better	Moderately better	Very much better

Please go on to the next page

This box to be completed by the clinic nurse or clinical research associate: Pt. Serial #: __________ Pt. Initials: ___ ___ ___

Please check to make sure you have answered all the questions

Please use the space below for any additional comments you may have:

____

____

____

____

____

Please fill in your initials to indicate that you have completed this questionnaire: ______________

Today's date (Year, Month, Day): ________________________________

Thank you.

This box to be completed by the clinic nurse or clinical research associate: Pt. Serial #: __________ Pt. Initials: ___ ___ ___

Eastern Cooperative Oncology Group (ECOG) FACT-P (version 2)

Quality of Life Questionnaire (PR.3)

Below is a list of statements that other people with your illness have said are important. By circling one number per line, please indicate how true each statement has been for you during the past 7 days.

During the past 7 days:

Circle one number

Physical Well-being	not at all	a little bit	some-what	quite a bit	very much
1. I have a lack of energy	0	1	2	3	4
2. I have nausea	0	1	2	3	4
3. I have trouble meeting the needs of my family	0	1	2	3	4
4. I have pain	0	1	2	3	4
5. I am bothered by side effects of treatment	0	1	2	3	4
6. In general, I feel sick	0	1	2	3	4
7. I am forced to spend time in bed	0	1	2	3	4

8. How much does your physical well-being affect your quality of life?

Not at all 0 1 2 3 4 5 6 7 8 9 10 Very much so

 Please go on to the next page

This box to be completed by the clinic nurse or clinical research associate: Pt. Serial #: __________ Pt. Initials: ___ ___ ___

During the past 7 days: Circle one number

Social/Family Well-Being	not at all	a little bit	some-what	quite a bit	very much
9. I feel distant from my friends	0	1	2	3	4
10. I get emotional support from my family	0	1	2	3	4
11. I get support from my friends and neighbours	0	1	2	3	4
12. My family has accepted my illness	0	1	2	3	4
13. Family communication about my illness is poor	0	1	2	3	4
If you have a spouse/partner, or are sexually active, please answer # 14-15. Otherwise go to # 16.					
14. I feel close to my partner (or main support)	0	1	2	3	4
15. I am satisfied with my sex life	0	1	2	3	4

16. How much does your social/family well-being affect your quality of life?

Not at all 0 1 2 3 4 5 6 7 8 9 10 Very much so

During the past 7 days: Circle one number

Relationship With Doctor	not at all	a little bit	some-what	quite a bit	very much
17. I have confidence in my doctor(s)	0	1	2	3	4
18. My doctor is available to answer my questions	0	1	2	3	4

19. How much does your relationship with the doctor affect your quality of life?

Not at all 0 1 2 3 4 5 6 7 8 9 10 Very much so

Please go on to the next page

During the past 7 days: Circle one number

Emotional Well-Being	not at all	a little bit	some-what	quite a bit	very much
20. I feel sad	0	1	2	3	4
21. I am proud of how I'm coping with my illness	0	1	2	3	4
22. I am losing hope in the fight against my illness	0	1	2	3	4
23. I feel nervous	0	1	2	3	4
24. I worry about dying	0	1	2	3	4

25. How much does your emotional well-being affect your quality of life?

Not at all 0 1 2 3 4 5 6 7 8 9 10 Very much so

During the past 7 days: Circle one number

Functional Well-Being	not at all	a little bit	some-what	quite a bit	very much
26. I am able to work (include work in home)	0	1	2	3	4
27. My work (including work in home) is fulfilling	0	1	2	3	4
28. I am able to enjoy life "in the moment"	0	1	2	3	4
29. I have accepted my illness	0	1	2	3	4
30. I am sleeping well	0	1	2	3	4
31. I am enjoying my usual leisure pursuits	0	1	2	3	4
32. I am content with the quality of my life right now	0	1	2	3	4

33. How much does your functional well-being affect your quality of life?

Not at all 0 1 2 3 4 5 6 7 8 9 10 Very much so

Please go on to the next page

This box to be completed by the clinic nurse or clinical research associate: Pt. Serial #: __________ Pt. Initials: ___ ___ ___

During the past 7 days: Circle one number

Additional Concerns	not at all	a little bit	some-what	quite a bit	very much
34. I am losing weight	0	1	2	3	4
35. I have a good appetite	0	1	2	3	4
36. I have aches and pains that bother me	0	1	2	3	4
37. I have certain areas of my body where I experience significant pain	0	1	2	3	4
38. My pain keeps me from doing things I want to do	0	1	2	3	4
39. I am satisfied with my present comfort level	0	1	2	3	4
40. I am able to feel like a man	0	1	2	3	4
41. I have trouble moving my bowels	0	1	2	3	4
42. I have difficulty urinating	0	1	2	3	4
43. I urinate more frequently than usual	0	1	2	3	4
44. My problems with urinating limit my activities	0	1	2	3	4
45. I am able to have and keep an erection	0	1	2	3	4

46. How much do these additional concerns affect your quality of life?

Not at all 0 1 2 3 4 5 6 7 8 9 10 Very much so

Please fill in your initials to indicate that you have completed this questionnaire: ______________

Today's date (Year, Month, Day): ________________________________

Thank you.

18.0 EXEMPLE DE FORMULAIRE DE CONSENTEMENT

ÉTUDE INTERGROUPE (GEC-INCC, CUOG, SWOG et MRC-UK) RANDOMISÉE DE PHASE III VISANT À COMPARER LE BLOCAGE TOTAL DE L'ACTION DES ANDROGÈNES EMPLOYÉ SEUL AVEC LE BLOCAGE TOTAL DES ANDROGÈNES COMBINÉ À UNE IRRADIATION PELVIENNE DANS LE TRAITEMENT DE L'ADÉNOCARCINOME CLINIQUE DE LA PROSTATE
GEC INCC : PR.3, SWOG : JPR.3, MC-UK : PR07

Objectif de l'étude

Mon médecin m'a informé que je souffre d'un cancer de la prostate. Les spécialistes ne s'entendent pas sur la meilleure façon de traiter ce cancer. Je suis conscient qu'il n'existe pas de traitement standard pour le cancer de la prostate à ce stade d'évolution. On a généralement recours à la radiothérapie, à l'hormonothérapie ou à une combinaison de ces traitements, et on obtient des résultats semblables quel que soit le traitement employé.

Afin de déterminer le meilleur traitement pour cette maladie, le NCIC Groupe des essais cliniques a élaboré la présente étude, et j'ai été invité à y participer. On m'a expliqué que l'étude vise à comparer deux formes de traitement pour ma maladie. Si je participe à l'étude, on m'assignera, purement au hasard, un des deux traitements suivants : une hormonothérapie employée seule ou une hormonothérapie combinée à une radiothérapie. Les probabilités que je reçoive l'un ou l'autre des traitements à l'étude sont égales. Je serai informé du traitement qui me sera assigné. Si on m'assigne l'hormonothérapie seule, je serai suivi de près et, si ma tumeur commence à se développer dans mon bassin mais qu'elle ne s'étend pas à d'autres parties du corps, on pourra alors me donner de la radiothérapie en plus de l'hormonothérapie.

Je comprends que l'objectif de l'étude est de déterminer quel des deux traitements est le plus efficace pour prolonger l'espérance de vie et assurer la meilleure qualité de vie possible pour les patients dont le cancer de la prostate a atteint ce stade d'évolution.

Quelque 1 200 personnes des États-Unis, du Canada et d’autres pays participeront à cette étude.

Traitements à l'étude

Tous les patients recevront de l'hormonothérapie. Il s'agira SOIT d'une combinaison d’un antiandrogène (flutamide, nilutamide ou bicalutamide) en comprimés et d'une antihormone appelée agoniste de la LH-RH (goséréline, leuprolide ou buséréline) en injections, SOIT une orchidectomie bilatérale (chirurgie). Je comprends que j'aurai à choisir entre ces deux thérapies hormonales - injections ou orchidectomie. L'orchidectomie est une intervention chirurgicale qui se pratique sous anesthésie générale et qui consiste à enlever les testicules. Je comprends que si je décide de recevoir l'agoniste de la LH-RH plutôt que de me faire enlever les testicules, je devrai me déplacer pour me faire donner une injection une fois par mois ou tous les deux mois ou tous les trois mois, pour le reste de ma vie ou jusqu’à ce que le traitement échoue. Quant aux comprimés d’antiandrogène, je devrai les prendre tous les jours aussi longtemps que mon médecin ne le prescrira.

Date de la version de cette formule ou date d’approbation par le CER : ___________	N^o série Pt NCIC GEC :_____

Je comprends que si je fais partie du groupe qui reçoit, en plus, de la radiothérapie, les traitements me seront donnés cinq fois par semaine pendant sept semaines (35 traitements). La zone irradiée comprendra la prostate et les ganglions lymphatiques qui l'entourent, à moins que ces derniers aient été examinés lors de la chirurgie et que cet examen ait démontré qu'ils n'étaient pas atteints; dans ce cas, la zone irradiée se limitera à la prostate.

Risques et effets secondaires possibles

Tous les traitements comportent des effets secondaires, et mon médecin me suivra de près afin de traiter tout problème qui pourrait apparaître.

Les risques et les effets secondaires communs à deux ou plus des agents à gonadolibérine utilisés au cours de cette étude comprennent les suivants :

Très probables (21 % ou plus) :
On m'a dit que traitement aux hormones pourrait produire les effets secondaires très probables suivants :

- impotence (incapacité d'avoir une érection) et baisse de la libido;
- bouffées de chaleur;
- nausées, vomissements;
- douleur.

Moins probables (5 à 20 %) :
On m'a dit que le traitement aux hormones pourrait produire les effets secondaires moins probables suivants :

- développement excessif des seins chez les hommes, qui peut inclure la sensibilité des seins;
- insomnie (difficulté à dormir);
- léthargie (sensation de fatigue);
- envie accrue d'uriner et besoin d'uriner plus souvent;
- altération de la vision nocturne;
- sensibilité à l'alcool;
- rapetissement des testicules;
- changements de la tension artérielle (hypotension ou hypertension) qu'on a rarement associés à des accidents vasculaires cérébraux (Buséréline);
- sensations de vertige qui peuvent inclure la pièce qui tourne;
- engourdissement et picotements dans les mains et les pieds;
- douleur locale, rougeur ou irritation aux points d'injection si je reçois des injections d'agonistes de la gonadolibérine);
- constipation;
- formation, dans les poumons, de cicatrices qui peuvent faire leur apparition chez des patients traités au Nilutamide qui cause de l'essoufflement et la toux. La plupart des patients se rétablissent lorsqu'ils cessent de prendre le médicament, mais il peut arriver très rarement que celui-ci cause la mort. Si un essoufflement nouveau fait son apparition ou si mon essoufflement s'aggrave, je le signalerai immédiatement à mon médecin;
- manque ou perte de force;
- maux de tête et vision floue;
- douleurs musculaires;
- douleurs articulaires;
- douleurs osseuses;
- changements des concentrations de lipides et de protéines dans le sérum;
- battement cardiaque irrégulier;
- changements de l'appétit.

Date de la version de cette formule ou date d'approbation par le CER : ____________	N° série Pt NCIC GEC :_____

Rares (1 à 4 %) :
On m'a dit que le traitement aux hormones pourrait produire les autres effets secondaires rares suivants :

- rétention d'un volume d'eau plus important dans les tissus;
- éruptions qui démangent;
- diarrhée;
- perte ou prise de poids;
- sensibilité du foie (jaunisse) – Si cela se produit, je devrai cesser de prendre les pilules antiandrogènes et communiquer avec mon médecin sur-le-champ;
- élévation des résultats de tests de fonction hépatique;
- toux;
- réaction de flambée de la maladie (douleur accrue dans les os, blocage des voies urinaires, compression de la moelle épinière) qui peut causer de la douleur, des picotements dans les bras et les jambes et des problèmes de mouvement et de sensation qui incluent rarement la paralysie;
- cardiopathie, y compris insuffisance cardiaque et crise cardiaque.

Risques et effets secondaires supplémentaires associés au LEUPROLIDE que l'on considère comme *médicalement importants* :

Moins probables (5 à 20%) :
- excès de sucre dans le sang (hyperglycémie);
- troubles neuromusculaires.

Rares (1 à 4 %) :
- changements de la vision;
- saignement dans le tractus gastro-intestinal (c.-à-d. l'estomac ou les intestins);
- perte de mémoire;
- perte de l'ouïe ou tintements dans les oreilles;
- réaction allergique, peut inclure le choc anaphylactique (anaphylaxie) dans les cas graves;
- fièvre.

On a rarement associé de *troubles pulmonaires* (y compris inflammation, pneumonie et accumulation de liquide) à l'usage du LEUPROLIDE.

Risques et effets secondaires supplémentaires associés à la GOSÉRÉLINE que l'on considère comme *médicalement importants* :

Rares (1 à 4%) :
- baisse de la fonction de la moelle osseuse qui peut réduire le nombre de cellules luttant contre les infections;
- syndrome ressemblant à celui de la grippe;
- infection;
- changements de la vision;
- fièvre;
- tumeur bénigne à l'hypophyse (adénome hypophysaire).

Risques et effets secondaires supplémentaires associés à la BUSÉRÉLINE qui sont considérés comme *médicalement importants* :

Date de la version de cette formule ou date d'approbation par le CER : ___________	N° série Pt NCIC GEC :_____

Moins probables (5 à 20 %) :
- dépression.

Rares (1 à 4 %) :
- changements de la vision;
- perte de mémoire;
- réaction allergique qui peut inclure le choc anaphylactique (anaphylaxie) dans les cas graves;
- tumeur bénigne à l'hypophyse (adénome hypophysaire);
- fièvre.

Baisse de la densité osseuse (perte osseuse) possible chez les patients traités par des agonistes de la gonadolibérine pendant longtemps.

On a rarement associé la *thromboembolie* (formation de caillots sanguins) aux agents antiandrogènes utilisés au cours de cette étude.

Pendant la radiothérapie :

Très probables (5 à 20 %) :
On m'a dit que le traitement aux hormones pourrait produire les effets secondaires très probables suivants :

- Diarrhée et irritation rectale qui peuvent se manifester sous forme d'une pression ou sensation de plénitude au rectum, inconfort et douleur à proximité de l'anus, irritation des hémorroïdes et saignements, moins probables.
- Irritation de la vessie qui pourrait me pousser à uriner plus souvent (à la fois le jour et la nuit) et à me précipiter vers la salle de bain lorsque j'ai envie d'uriner.
- Rougeurs et sensibilité légères de la peau dans la région irradiée – On m'a dit que c'est rarement grave.
- Fatigue et nausées (vomissements rares) – On m'a dit que ces symptômes sont rarement graves.
- Impotence (incapacité d'avoir une érection).

<u>Après la radiothérapie :</u>

Rares (1 à 4 %) :
Je comprends que des patients peuvent ressentir des effets secondaires persistants graves rares de la radiothérapie, dont les suivants :

- Diarrhée et irritation rectale qui peuvent se manifester sous forme d'une pression ou sensation de plénitude au rectum, inconfort et douleur à proximité de l'anus, irritation des hémorroïdes et saignements, moins probables. Un ulcère rectal peut très rarement faire son apparition et causer de la douleur, du saignement et l'expulsion de mucus avec les selles.
- Irritation de la vessie qui pourrait me pousser à uriner plus souvent (à la fois le jour et la nuit) et à me précipiter vers la salle de bain lorsque j'ai envie d'uriner.
- Impotence (incapacité d'avoir une érection).
- Rétrécissement du col de la vessie – Je comprends que cet effet peut me causer de la difficulté à uriner et pourrait m'obliger à subir une intervention chirurgicale.

De plus, il existe toujours le risque que surviennent des effets secondaires très rares ou inconnus jusqu'à présent. J'ai discuté avec mon médecin des effets secondaires et je réalise que plusieurs d'entre eux sont irréversibles.

Je comprends qu'on ne connaît pas suffisamment ces traitements pour savoir s'ils risquent de nuire à un enfant que je concevrais pendant que je participe à l'étude. Par conséquent, il faudra que mes partenaires sexuels ou moi-même prenions des moyens de contraception efficaces aussi longtemps que je participerai à l'étude.

Mon médecin me suivra de près afin de déceler les effets secondaires. Mon médecin pourra me prescrire des médicaments (ou espacer les traitements), de façon à ce que les effets secondaires soient tolérables.

Si on découvrait de nouveaux effets secondaires ou si on obtenait de nouvelles informations sur mon traitement au cours de la présente recherche, j'en serais informé.

<u>Avantages potentiels</u>

On m'a expliqué qu'on ignore si je retirerai un avantage quelconque de ma participation à l'étude.

Je comprends que la présente étude pourrait démontrer que l'hormonothérapie employée seule est un traitement aussi efficace que l'hormonothérapie combinée avec la radiothérapie. Après plusieurs années de suivi, il sera peut-être possible de déterminer si l'hormonothérapie employée seule occasionne moins de complications et d'effets secondaires à long terme, ou si l'hormonothérapie combinée avec la radiothérapie cause plus de complications et d'effets secondaires.

On m'a expliqué que le traitement serait arrêté si ma maladie progressait, si les effets secondaires devenaient très graves, si de nouvelles informations indiquaient que le traitement que je reçois n'est pas dans mon meilleur intérêt, si mon médecin arrivait à la conclusion que ce traitement n'est plus dans mon meilleur intérêt, ou si le commanditaire mettait fin à l'étude. On discuterait alors des prochaines étapes de mon traitement.

Date de la version de cette formule ou date d'approbation par le CER : ____________	N° série Pt NCIC GEC :_____

Traitements de rechange

On m'a expliqué que si je ne participe pas à l'étude, les principaux traitements de rechange qu'on pourrait envisager dans mon cas sont la radiothérapie seule, une autre hormonothérapie ou une combinaison de ces traitements. Mon médecin a discuté avec moi des avantages potentiels et des effets secondaires possibles de ces différents traitements.

Examens et questionnaire

Je comprends que pendant ma participation à l'étude, je devrai me soumettre à un certain nombre d'examens (analyses de sang, radiographies). Ces examens sont du même type que ceux que j'aurais à subir si je ne participais pas à l'étude, mais ils risquent d'être faits plus fréquemment qu'à l'habitude, surtout pendant les premières phases de l'étude.

Les aiguilles utilisées pour prélever du sang ou pour injecter les substances nécessaires aux examens radiologiques peuvent provoquer un inconfort. Un hématome ou, ce qui est rare, une infection peut faire son apparition au point de ponction à l'aiguille.

Je comprends qu'à des intervalles définis de l'étude, on me demandera de remplir un questionnaire sur la qualité de vie conçu pour évaluer comment je me sens et comment je tolère le traitement. J'aurai besoin de 15 à 20 minutes pour le remplir. Je suis conscient que ce questionnaire constitue une partie importante de l'étude.

Confidentialité et accès aux dossiers

Des représentants qualifiés des organisations suivantes peuvent inspecter mes dossiers médicaux ou ceux de l'étude et en recevoir de l'information pour des fins d'assurance de la qualité et d'analyse des données:

- le NCIC Groupe des essais cliniques (NCIC GEC), le groupe de recherche qui coordonne l'étude;
- le comité de l'éthique de la recherche qui supervise la conduite éthique de cette étude dans votre hôpital/clinique;
- le Southwest Oncology Group;
- le MRC-UK;
- le National Cancer Institute des États-Unis;
- la Food and Drug Administration des États-Unis (parce qu'elle supervise l'utilisation des médicaments aux É.-U.);
- d'autres organismes de réglementation (parce qu'ils supervisent l'utilisation des médicaments dans d'autres pays).

Les organisations mentionnées ci-dessus maintiendront la confidentialité des renseignements qu'elles voient ou reçoivent à mon sujet, dans la mesure où les lois pertinentes le permettent.

Étant donné l'importance des résultats à long terme de cette étude, la signature du présent formulaire constitue une autorisation à vie aux organisations mentionnées ci-dessus de consulter mes dossiers médicaux en ce qui a trait à mon cancer et au traitement de ce dernier.

Aucun dossier mentionnant mon nom ne sera fourni à qui que ce soit, sauf aux chercheurs (chercheuses) participant à l'étude. Je ne serai identifié personnellement dans aucun rapport rédigé à la suite de l'étude.

MODIFIÉ : 2008-MAY-08

Coûts

Je ne toucherai aucun paiement pour participer à l'étude. La participation à cette étude pourrait m'imposer des frais.

En cas d'effets secondaires ou de traumatismes reliés à la recherche, mon médecin me dispensera des soins médicaux, ou l'on m'aiguillera vers les soins médicaux nécessaires.

Participation volontaire

J'ai discuté de l'information ci-dessus avec mon médecin, et il (elle) a répondu à toutes mes questions concernant mon traitement. Je comprends que ma participation à la présente étude est tout à fait volontaire, et que je serai libre de m'en retirer en tout temps. Si je choisis de ne pas participer à l'étude, ou de m'en retirer, mon médecin continuera à me soigner en ayant recours aux meilleurs traitements disponibles.

Date de la version de cette formule ou date d'approbation par le CER : ____________	N° série Pt NCIC GEC :_____

Je comprends aussi que si j'ai quelque question que ce soit concernant ma participation à l'étude, je peux en tout temps rencontrer le médecin qui est le principal responsable de l'étude. J'ai reçu un exemplaire du présent formulaire de consentement.

Message aux patients participant dans un des établissements associés au SWOG

En cas de blessure ou de complication résultant de ma participation à la présente étude, on mettra à ma disposition les traitements appropriés. Cependant, je ne recevrai pas automatiquement un remboursement de mes frais médicaux, ni une autre indemnité.

Pour plus d'information sur la recherche ou sur les risques ou blessures reliés à la recherche, je peux communiquer avec le docteur ______________, le chercheur (la chercheuse) responsable, au numéro (tél.)_____________. De plus, je peux appeler ____________________, au numéro (tél.) _____________, afin d'obtenir de l'information sur les droits des patients participant à des études.

Voici le nom d'une personne qui n'est aucunement reliée à l'étude et avec qui je peux discuter des questions relatives à ma participation à l'étude :

___________________________________ ____________________

Nom Téléphone

Le chercheur (La chercheuse) principal(e) de l'étude au centre où je suis suivi est :

___________________________________ ____________________

Nom Téléphone

Ma signature au bas du présent formulaire indique que j'accepte de participer à l'étude.

___________________________________ ____________________

Signature du patient Date

___________________________________ ____________________

Signature du chercheur (de la chercheuse) Date

A-t-on aidé le(la) patient(e) d'une des façons indiquées ci-dessous pendant le processus visant à obtenir le consentement?

☐ *Oui* ☐ *Non*

Si oui, veuillez cocher la case pertinente et remplir l'espace réservé à la signature ci-dessous :

☐ *On a lu le formulaire de consentement au(à la) patient(e) et la personne qui appose sa signature ci-dessous atteste qu'on a expliqué précisément l'étude au(à la) patient(e), qui semble l'avoir comprise.*

Réservé aux sites canadiens :

☐ La personne qui appose sa signature ci-dessous a fait fonction d'interprète pour le(la) patient(e) au cours du processus visant a obtenir le consentement.

_________________________________ _________________________

Signature de la personne qui a participé à la discussion sur le consentement **Date**

Date de la version de cette formule ou date d'approbation par le CER : ____________	N^{o} série Pt NCIC GEC :_____
